# Interdependence, bonding and support are associated with improved mental wellbeing following an outdoor team challenge

**DOI:** 10.1111/aphw.12351

**Published:** 2022-02-28

**Authors:** Emma Cohen, Arran J. Davis, Jacob Taylor

**Affiliations:** ^1^ Social Body Lab, Institute of Human Sciences, School of Anthropology and Museum Ethnography University of Oxford Oxford UK; ^2^ Wadham College Oxford UK

**Keywords:** outdoor learning, social behavior, social relationships, social support, team challenge, wellbeing

## Abstract

Social relationships and mental health are functionally integrated throughout the lifespan. Although recent laboratory‐based research has begun to reveal psychological pathways linking social interaction, interdependence, bonding and wellbeing, more evidence is needed to integrate and understand the potential significance of these accounts for real‐world events and interventions. In a questionnaire‐based, repeated measures design, we measured the wellbeing of 13‐ to 19‐year‐old participants (*n* = 226) in the Ten Tors Challenge (United Kingdom) 7–10 days before (T1) and after (T4) the event. Immediately before (T2) and after (T3) the event, we administered measures of team bonding, perceived and experienced interdependence, perceived and received support, physical pain and fatigue, and performance satisfaction. There was a significant increase in participants' wellbeing (pre‐to‐post event). Post‐event social bonding and performance satisfaction positively predicted the wellbeing increase. Bonding was, in turn, positively predicted by experienced interdependence, received support, pain and fatigue, and the sense of having done better as a team than expected. Results provide novel field‐based evidence on the associations between meaningful bonds of mutual reliance in a challenging team event and adolescent wellbeing. Team challenge events potentially offer effective contexts for forging social interactions, interdependencies, and bonds that can support mental and physical health.

## INTRODUCTION

The promotion of adolescent mental health is a major global public health priority. Mental health conditions account for 16% of the global burden of disease and injury in 10–19 year‐olds, and depression is one of the leading causes of illness among adolescents (World Health Organization, 2020). There are established links between social relationships and mental health throughout the lifespan. In the context of concerns about the long‐term impact of COVID‐19 social restrictions on child and adolescent mental health, a recent rapid review of 83 articles (*n* = 51,576) identified social isolation and loneliness as risk factors for depression and anxiety up to 9 years later among previously healthy children and adolescents (Loades et al., 2020). Understanding how social interactions and relationships can promote health and prevent health decline, particularly through youth events and interventions, is critically important for orienting public health efforts to offer preventative support.

A rapidly growing literature has developed over recent decades on the importance of good quality social relationships for mental and physical health (Holt‐Lunstad & Uchino, 2008). Close, affiliative, supportive social bonds buffer stress and illness and are a fundamental basis of perceived meaning and purpose in life (Uchino et al., 1996). Relationships of mutual affection are cultivated through successful joint engagement in and commitment to cooperative projects (Castro & Pacherie, 2020) and are the evolutionary cradle of cooperation on a larger scale than is observed in any other primate. Their integral connections with mental and physical health are thought to derive from the fitness benefits of group belonging and the fitness harms of solitary isolation (Baumeister & Leary, 1995; Dunbar, 2018). This perspective brings into sharp focus the potential role of mutual interdependence and social support in the context of meaningful joint activities for forging and sustaining health‐promoting social bonds (Gerpott et al., 2018).

How do social interdependence, support, and bonding in the context of meaningful joint activities influence wellbeing? Although considerable research has examined the negative health effects of social isolation and the growing problem of loneliness (Cacioppo & Cacioppo, 2018; Hawkley & Cacioppo, 2010), studies on the *opposite of loneliness*, or social bonding, and on associations among joint activity, interdependence and support, social belonging, and wellbeing (including flourishing or thriving) have been more piecemeal and dispersed across the psychological, behavioral, and health sciences. Of particular relevance are investigations into the psychological and behavioral factors contributing to perceived social bonding and the significance of social bonding and support for wellbeing and health. Recent theoretical and experimental research has identified specific features of social interaction, such as coordination and interdependence that foster and sustain health‐promoting social bonds. For example, there is growing evidence of causal associations and pathways linking joint activity and social bonding (Wolf et al., 2016), behavioral interdependence and group identification, bonding and cooperation (Michael et al., 2016; Tunçgenç & Cohen, 2016), and (shared) physical pain or fatigue and social bonding and support (Bastian et al., 2014). These investigations are theoretically situated within approaches to the evolution, development, and psychology of human cooperative affiliation. Proceeding largely independently from this work is a large body of research on the importance of cooperative social relationships for wellbeing (Hornstein et al., 2016; Jetten et al., 2012). Social psychological approaches have, in particular, explored the importance of social identities and group life for health (Cruwys et al., 2014). Multidimensional approaches to wellbeing—understood not just as the absence of illness but as optimal psychosocial functioning across multiple domains—consistently emphasize the importance of social relationships for flourishing (e.g., Butler & Kern, 2016).

Integrative theoretical approaches have been proposed, such as Baumeister and Leary's (1995) influential social motivational account of humans' “need to belong,” Beckes and Coan's (2011) and Coan and Sbarra's (2015) ecological social baseline theory and later extensions to relational affect (Sbarra & Coan, 2018), and Aktipis et al.'s (2018) evolutionary “fitness interdependence” framework. Although the focus of each approach is distinct, these integrative frameworks commonly point to humans' interdependent social ecology as a default evolutionary and developmental context in which cognitive, affective, and physiological function emerges and adapts. From birth and throughout life, human social situations are characterized by distinct kinds of cooperative interdependence, including mutual reliance in the pursuit of optimal individual and group outcomes (Gerpott et al., 2018). In close relationships, cooperative interdependence entails significant exchange of resources, such as aid, support, and protection, and is sustained via a range of intense sentiments, including feelings of affection, love, closeness, attachment, safety, warmth, empathy, and kinship (Aktipis et al., 2018). Access to close cooperative relationships also alters perceptions of and emotional responses to environmental stimuli, including potential threats, which in turn modulate affective and physiological responses and actions (e.g., withdrawal or approach; Sbarra & Coan, 2018). This research therefore suggests that the evolutionary‐developmental necessity of cooperative interdependence for surviving and thriving links cooperative sociality to health and wellbeing via a range of affective (and associated, e.g., physiological) pathways.

These approaches offer an overarching view of the links among the evolution of cooperative interdependence, proximate psychological motivations to bond and belong, and relational affect and wellbeing. Recent controlled psychological and behavioral studies have explored how behavior and emotions vary across different types of interdependent situations (e.g., happiness or anger, depending on whether the situation is characterized by high or low conflict) (Gerpott et al., 2018), how social bonds are sustained through interdependent activity, or “social grooming” (e.g., Tarr et al., 2015, 2018; Tunçgenç & Cohen, 2016), and how social—particularly group—identities and interpersonal kindness interventions can be leveraged to improve health and wellbeing outcomes (Haslam et al., 2019; Rowland & Curry, 2019). These studies have revealed positive health and wellbeing effects of having strong social identities, perceiving/receiving social support, and giving social support, such as in performing kindness activities. However, controlled research that combines key insights across frameworks to investigate associations among subjective experiences of mutual interdependence (e.g., support or help given *and* received/perceived), social bonding (a distinct but potentially overlapping construct from/with social identification), and mental wellbeing (including both alleviation and protection from illness and psychological flourishing/thriving) in the context of a discrete, self‐motivated, real‐world activity is lacking.

The current study draws from complementary accounts of human cooperation and wellbeing to examine associations among subjective assessments of *interdependence*, taken to refer to mutual reliance and support in the pursuit of joint goals (Gerpott et al., 2018), *social support*, referring to assistance provided by others (Cohen & Wills, 1985), *social bonding*, referring to relationships of social closeness and companionship (Dunbar & Shultz, 2010), and *mental wellbeing* across multiple dimensions (e.g., positive affect and psychological functioning; Tennant et al., 2007). Taken together, these accounts suggest that interdependence and social support in joint activity are associated with emotions, perceptions, and behaviors that enhance perceived social bonding and (directly and in turn) facilitate the successful pursuit of demanding individual and joint goals (Balliet et al., 2016; Gerpott et al., 2018). This, in turn, enhances positive affect, psychosocial functioning, and health via multiple direct and indirect pathways (Cohen, 2004; Slavich, 2020).

The specific aim of the current research is to establish preliminary evidence for the proposed associations among subjective experiences and appraisals of interdependence, support, performance, bonding and mental wellbeing among adolescent peers in a meaningful real‐world activity. In particular, we examine effects of perceived and experienced interdependence on bonding and effects of bonding and performance satisfaction on wellbeing. The work extends existing experimental and observational research that addresses these links in a more piecemeal way. Although experimental studies can offer high control, combining these elements (i.e., challenging group situations characterized by interdependent mutual support and highly valued, meaningful goals that can potentially impact social bonds and wellbeing) presents challenges for laboratory‐based research. The frequent co‐occurrence of these elements in highly structured, real‐world youth events, programs, and interventions can help advance our understanding of their interconnections in a naturalistic setting while maintaining a high level of methodological control (cf. Singh et al., 2020).

One such event is the Ten Tors Challenge (TTC), a major youth challenge held annually in the United Kingdom. This team‐based event offers an ideal natural laboratory setting in which to investigate effects of perceived interdependence, social support, and performance success on social bonds among team members and how these, in turn, influence mental wellbeing (further described below). Recent research describes a rapid increase in mental health conditions among UK adolescents in the past decade (Gunnell et al., 2018). The escalating demand on struggling mental health services indicates the profound necessity of evidence‐based preventative approaches that can support young people's ability to thrive (Lennon, 2021). Schools are widely recognized for their role in supporting students' mental health, and the commitment to mental health education has been expanded recently in the national curriculum at all levels.^1^ Across UK schools, there is also a long‐standing tradition of outdoor education, focused on experiential learning through play, adventure, risk‐taking, and team‐work (Williams & Scott, 2019). However, despite pervasive themes related to wellbeing in the relevant educational literature on outdoor learning (e.g., around meaningful experience, enjoyment, engagement, self‐confidence, mood, and relationships), potential links to positive mental health have not been systematically explored or quantified (Williams & Scott, 2019).

### Hypotheses

The current study contributes new data on the potential importance of team challenge events for mental wellbeing and begins to test theory‐driven hypotheses derived from the broader literature on interdependence, social bonding, and wellbeing. Here, we focus primarily on subjective mental wellbeing, which relates to the subjective experience of positive affect and psychological functioning, though our contribution can be situated within a broader integrative health framework. The TTC is an intensely challenging event, which also allowed us to investigate links between pre‐event bonding and subjective experiences of pain and fatigue during the event (Davis & Cohen, 2018; Davis, Crittenden, & Cohen, 2021; Davis, MacCarron, & Cohen, 2021) as well as effects of pain and fatigue on post‐event bonding (Bastian et al., 2014; Kavanagh et al., 2019).

Specifically, we hypothesized as follows: H1: an increase in wellbeing (pre‐to‐post event); H2: wellbeing increase would be positively predicted by team bonding and performance satisfaction; H3: bonding would be positively predicted by physical discomfort (pain and fatigue), received support, experienced interdependence, and team performance relative to expectations (i.e., the feeling of succeeding together against the odds); H4: physical discomfort would be negatively predicted by pre‐event bonding and perceived support. Additional exploratory analyses investigated E1: pre‐event (T2) associations of perceived interdependence and support on bonding; E2: effect of post‐event bonding on performance satisfaction; E3: effects of perceived support on collective efficacy and pre‐event perceptions of the event as threatening; E4: interactions between physical discomfort and received support and interdependence on bonding. All measures were assessed using self‐report questionnaires.

## METHOD

### Participants

A total of 239 participants were recruited via communications on the TTC website and from the organizers to all team managers (i.e., designated adult responsible for each team); all TTC participants were invited, and those who gave consent were included in the research (there were no exclusions). All were participants in TTC in 2017, and all data were collected between April 26, 2017, and May 17, 2017. TTC teams and participants are largely convened within local schools, scout, and cadet organizations as well as trekking, rambling and expedition societies. Thirteen participants who gave consent did not complete any questionnaires. The final number for analysis was 226. The mean age of participants was 15 years, 9 months (*SD* = 1 year, 3 months, range = 13 years, 0 months to 19 years, 7 months), and 91 of the participants were female (40.27%). No further demographic data were collected for analysis.

The research was approved by the Departmental Research Ethics Committee of the School of Anthropology and Museum Ethnography, University of Oxford, and informed consent was obtained from participants and parents prior to participation. TTC organizers, team managers, parents, and prospective participants were informed about the purpose of the research (“to gain a better understanding of the psychological links between group exercise challenges, team bonding and wellbeing among participants”); that it would entail four online surveys, that participation in any or all of the surveys was voluntary, and that participants could withdraw from the research at any time without consequences for their TTC participation. Team managers who agreed to their team's involvement provided parents/caregivers with an electronic link to the online information and consent form, which parents were requested to discuss with their children. Parents provided participants' contact details within their consent, and the researchers then contacted participants with further details of the study, including the information sheet and a link to the first questionnaire. Researcher contact details were provided for any queries.

### The TTT event

The TTC event is a 2‐day expedition held annually on a weekend in May within Dartmoor National Park, United Kingdom. It is organized and run by the British Armed Forces alongside the emergency services and many voluntary organizations and individuals. Each team of six participants must visit 10 specified manned locations, requiring a walk of 35, 45, or 55 miles. A team must be self‐sufficient, camp on the moor overnight, and complete the distance between 07:00 on Saturday and 17:00 on Sunday. The 2017 event had a total of 410 teams (2,460 participants): 261 (63.66%) entered the 35‐mile challenge, 98 (23.90%) entered the 45‐mile challenge, and 51 (12.44%) entered the 55‐mile challenge. Our participant sample included 128 35‐mile, 57 45‐mile, and 41 55‐mile challengers (56.65%, 25.22%, and 18.14%, respectively), providing a relatively representative spread across the three distance categories.

### Procedure

Four questionnaires were administered: 7–10 days before the event (T1), the evening before the event (T2), immediately after the event finish line but prior to the route‐exit (T3), and 7–10 days after the event (T4). All questionnaires were administered following informed consent online via http://www.onlinesurveys.ac.uk. Participants received a link via email to the questionnaires at T1 and T4 and were asked to complete the questionnaires in their own time at home. Questionnaires at T2 and T3 were administered at the TTC venue in a special‐purpose laboratory tent with internet‐enabled computer terminals. Participants visited the tent on the Friday evening prior to the event start and completed the online T2 questionnaire. The T3 questionnaire was administered immediately on crossing the event finish line on the following Sunday. Hard copy T3 questionnaires were provided to participants who had withdrawn from the challenge to complete at the “fall out center.”^2^ Questionnaires at T1, T3, and T4 took approximately 5–10 min and at T2 approximately 15–20 min to complete.

### Measures and data reduction

Table 1 contains details of all measures used to test hypotheses and descriptive results. A confirmatory factor analysis (CFA) was carried out on all variables that related to pre‐validated measures (e.g., mood and wellbeing) to check factor loadings for these measures against those previously published (Matsunaga, 2010). For measures that were not previously validated, we conducted principal component analyses (PCAs) on the relevant variables and used the resulting statistical constructs as measures for subsequent analyses. PCA was also used when factor loadings from CFAs did not align with previously validated measures; components for the variables shown to load onto each relevant factor were created using PCA. Full results can be found in Section S2; all variables can be assumed to have measurement invariance, unless noted otherwise (see Section S3 for full results of measurement invariance analyses).

**TABLE 1:** Measures and descriptive results

Variable	Measure	Sample question	Response scale	T1 mean (*SD*)	T2 mean (*SD*)	T3 mean (*SD*)	T4 mean (*SD*)
Wellbeing	Warwick–Edinburgh Mental Wellbeing Scale	e.g., I've been feeling good about myself	None of the time (1)–all of the time (5)	3.773 (0.498)	—	—	3.846 (0.516)
Behavioral interdependence	Group Identification Scale	e.g., all members need to contribute to achieve the team's goals	Strongly disagree (1)–strongly agree (7)	—	5.942 (1.024)	—	—
Mood	Brunel Mood Scale
Anger		e.g., bitter	Not at all (1)–extremely (5)	—	0.106 (0.251)	0.272 (0.566)	—
Confusion		e.g., muddled	Not at all (1)–extremely (5)	—	0.332 (0.397)	0.348 (0.538)	—
Depression		e.g., downhearted	Not at all (1)–extremely (5)	—	0.118 (0.281)	0.256 (0.579)	—
Fatigue		e.g., tired	Not at all (1)–extremely (5)	—	0.713 (0.592)	2.783 (0.975)	—
Tension		e.g., worried	Not at all (1)–extremely (5)	—	1.109 (0.821)	0.232 (0.433)	—
Vigor		e.g., energetic	Not at all (1)–extremely (5)	—	2.404 (0.731)	1.967 (0.949)	—
Perceptions of relationship	Perceptions of relationship						
Closeness		e.g., how much do you enjoy spending time with your team members?	Not at all (1)–very much (7)	—	5.767 (0.803)	—	5.374 (1.037)
Similarity		e.g., my team members and I have a similar outlook on life	Not at all (1)–very much (7)	—	5.064 (1.088)	—	4.953 (1.204)
Everyday centrality		e.g., how often do you talk to your team members?	Not at all (1)–very much (7)	—	5.108 (1.207)	—	4.600 (1.515)
Bonding					5.343 (0.786)	5.652 (0.842)	5.046 (1.072)
Connected	Single question	How connected do you feel to the other members of your team?	Not at all (1)–very much (7)	—	5.410 (1.164)	5.964 (1.158)	5.205 (1.319)
Bonded	Single question	How bonded do you feel to the other members of your team?	Not at all (1)–very much (7)	—	5.627 (1.054)	6.051 (1.058)	5.416 (1.330)
Committed	Single question	How committed do you feel to the other members of your team?	Not at all (1)–very much (7)	—	6.075 (0.991)	6.142 (1.040)	5.453 (1.369)
Identity fusion	The pictorial measure of fusion	Please indicate which of the following images best describes your relationship to your team	1–5, where 1 shows separate circles (representing self and team, respectively) and 5 shows completely overlapping circles.	—	4.261 (0.817)	4.464 (0.787)	4.112 (0.975)
Perceived support	Perceived Availability of Support in Sport Questionnaire (T2)						
Emotional		e.g., … show concern for you?	Not at all (0)–very much (6)	—	5.106 (0.849)	—	—
Esteem		e.g., … boost your sense of competence?	Not at all (0)–very much (6)	—	5.128 (0.824)	—	—
Received support	Athletes' Received Support Questionnaire (T3)^a^
Emotional		e.g., … comfort you?	Not at all (1)–very much (5)	—	3.961 (0.819)	—	—
Esteem		e.g., … encourage you?	Not at all (1)–very much (5)	—	4.075 (0.835)	—	—
Stress appraisal	Stress Appraisal Measure						
Threat (emotion)		e.g., … anxious.	Not at all (1)–extremely (5)	—	2.286 (0.906)	—	—
Threat (outcome)		e.g., … the outcome will be negative.	Not at all (1)–extremely (5)	—	1.260 (0.451)	—	—
Challenge		e.g., … it will have a positive impact on you	Not at all (1)–extremely (5)	—	4.539 (0.493)	—	—
Centrality		e.g., … it will have important consequences for you	Not at all (1)–extremely (5)	—	2.975 (0.804)	—	—
Collective efficacy	Collective Efficacy for Sports (short version)	e.g., do you think your team is effectively prepared for the activities?	Not at all (0)–very much (10)	—	7.466 (1.532)	—	—
Actual interdependence	Single items on help and need	Thinking about your team as a whole …		—	—	6.091 (1.532)	—
Help		… how much did you need each other?	Not at all (1)–very much (7)	—	—	6.026 (1.139)	—
Need		… how much did you help each other?	Not at all (1)–very much (7)	—	—	6.155 (1.044)	—
Performance satisfaction				—	—	4.507 (0.641)	—
Individual	Single item	How satisfied are you with your individual performance?	Not at all (1)–very much (5)	—	—	4.426 (0.840)	—
Team	Single item	How satisfied are you with the performance of your team?	Not at all (1)–very much (5)	—	—	4.589 (0.684)	—
Performance relative to expectation				—	—	6.734 (1.694)	—
Individual	Single item	How does your individual performance compare to your expectations before the event?	1 (much worse than expected)–5 (as expected)–10 (much better than expected)	—	—	6.553 (2.006)	—
Team	Single item	How does the performance of your team compare to your expectations before the event?	1 (much worse than expected)–5 (as expected)–10 (much better than expected)	—	—	6.914 (1.781)	—
Discomfort				—	—	6.508 (1.919)	—
Fatigue	Single item	How much fatigue did you experience overall?	None at all (1)–extremely high (10)	—	—	6.508 (2.372)	—
Pain	Single item	How much pain did you experience overall?	None at all (1)–extremely high (10)	—	—	6.508 (1.950)	—
Effort	Single item	How much effort did you put in overall?	None at all (1)–extremely high (10)	—	—	9.223 (0.998)	—

^a^ Response options were adapted from the original published scale (1–5: Not at all, once or twice, three or four times, five or six times, seven or more times).

Wellbeing (T1 and T4) was measured using the Warwick–Edinburgh Mental Wellbeing Scale (WEMWBS; Tennant et al., 2007). The WEMWBS is a 14‐item measure of mental wellbeing that has been validated for use among teenage school students in the United Kingdom, with a Cronbach's α of .87, indicating good internal consistency (Clarke et al., 2011). Following a CFA, three items were dropped due to below‐threshold factor loadings (see Section S2.1.9 for details). The resulting wellbeing factor had good internal consistency (Cronbach's α at T1 = .84 and at T4 = .88; see Section S2 for McDonald's *ω* values for all relevant measures).

Bonding (T2–T4) was measured using a pictorial measure of identity fusion (Swann et al., 2009) and three items that assessed how connected, bonded, and committed participants felt to the other members of their team. Items were drawn from previous experimental studies on social bonding in joint physical activities (e.g., Tarr et al., 2015, 2018). A PCA at each time point explained between 60.39% and 72.69% of the variance. Cronbach's α's at T2–T4 were .78, .84, and .87, respectively, indicating good internal consistency for the bonding component across time points (see Sections S2.2.1–S2.2.3 for details).

Emotional and esteem subscales of the Perceived Availability of Support in Sport Questionnaire (PASS‐Q; Freeman et al., 2011) and the Athletes' Received Support Questionnaire (ARSQ; Freeman et al., 2014; T3) were used to measure perceived support and received support at T2 and T3, respectively. Both scales are widely used to measure perceived and received social support in sports contexts, including with adolescents (e.g., Abadi & Gill, 2020; Stanger et al., 2018). Perceived support refers to one's subjective assessment of the hypothetical availability of support if needed. Received support refers to one's subjective assessment of assistance actually given. Whereas emotional support relates to love, care, comfort, and security derived from others, esteem support reflects encouragement and bolstering of self‐confidence, self‐esteem, and sense of competence. A CFA was run on the 10 questions making up the emotional and esteem dimensions and results supported the two‐factor model at T2 and T3 (see Sections S2.1.3 and S2.1.4 for details). The emotional and esteem subscales of the PASS‐Q (T2) had good internal consistency (Cronbach's α = .87 and .88, respectively), as did those of the ARSQ (T3; Cronbach's α = .84 and .88, respectively).

The behavioral interdependence subscale of the Group Identification Scale (Henry et al., 1999) was used to measure subjective perceptions of within‐team interdependence at T2. The subscale consists of four items that reflect sense of within‐group reliance and common fate and that have been linked to group identification, cohesion and cooperation (Henry et al., 1999). A CFA on the four items supported the single factor model. However, one reverse‐coded item was dropped due to low factor loading (see Section S2.1.11 for details). The Cronbach's α for the three‐item factor was .55, which falls well below the levels reported by Henry et al. (1999; .80 and .83 for two separate US student samples), and results are therefore interpreted with caution.

Experienced interdependence (T3) was measured using two novel items assessing the degree to which participants needed and helped each other. One component was extracted following a PCA (see Section S2.2.6 for details), which explained 80.28% of the variance, and the two items had a relatively high correlation (*r* = .61).

Two novel items measured subjective experiences of pain and fatigue during the event (measures taken at T3), and these were combined into a single component, physical discomfort, following PCA (see Section S2.2.4 for details), which explained 78.61% of the variance (*r* = .57). Performance satisfaction (T3) was measured using two novel items, assessing how satisfied participants were with their individual performance and the performance of their team. The two items were combined into a single component following PCA (see Section S2.2.5 for details), explaining 80.02% of the variance (*r* = .60). In addition to links between performance satisfaction and wellbeing, we specifically predicted that performing *better than expected* (i.e., the feeling of succeeding together against the odds) would predict team bonding, controlling for individual performance against expectations. Two further novel questions assessed how participants' perceptions of their (individual/team) performance compared with their expectations before the event and were used independently in analyses.

In addition to our main hypotheses, we also explored effects of perceived esteem and emotional support on collective efficacy at T2 (Collective Efficacy for Sports Scale; Páez et al., 2015; Zumeta et al., 2016) and pre‐event (T2) perceptions of challenge and threat (Stress Appraisal Measure [SAM]; Peacock & Wong, 1990). The four‐item measure of collective efficacy assesses an individual's perceived efficacy of their group to perform a sports activity. A CFA (see Section S2.1.2 for details) on the four items supported the single factor model, which had good internal consistency (Cronbach's α = .79). The dimensions of challenge and threat from the SAM each comprise four questions that assess positive (anticipation of gain/growth) and negative (potential for harm/loss) appraisals, respectively, of a potentially stressful situation. A CFA (see Section S2.1.1) suggested a four‐factor model for the SAM, with a single challenge factor (Cronbach's α = .71), a single centrality factor (Cronbach's α = .70), and two factors related to threat. Two components relating to threat were therefore extracted using PCA, which we have called threat emotion (Cronbach's α = .67) and threat outcome (Cronbach's α = .78), combining items that reflect perceptions of threat/anxiety and outcome/impact, respectively. Centrality—or perceived importance of the event—was included for descriptive reference but was not used in tests of hypotheses.

As this is the first time the TTC has been the focus of academic research, further measures aimed to capture a richer descriptive picture of participants' experiences and team relationships. These include the 24‐item Brunel Mood Scale (Terry et al., 1999; T2 and T3, dimensions of anger, confusion, depression, fatigue, tension, and vigor), the 15‐item Perceptions of Relationship Scale (Vangelisti & Caughlin, 1997; T2 and T4), and a question about level of effort (T3). The Brunel Mood Scale is a multidimensional mood scale that was originally developed for use with adolescents. Results of a CFA supported the original six‐factor model. With the exception of anger at T2 (Cronbach's α = .55), there was good internal consistency for the final factors at the two time points (for full details, including item exclusions, see Sections S2.1.5 and S2.1.6). The Perceptions of Relationship Scale was originally developed to measure dimensions of psychological closeness, similarity, and everyday centrality within the context of family relationships. As the item wording does not assume a particular relationship domain, it was readily generalizable to the current study context. Internal consistency was high for the three factors across the two time points (for full details, including item exclusions, see Sections S2.1.7 and S2.1.8).

To further understand participants' experiences and team relationships, mean difference tests were applied to factors from both the Brunel Mood Scale and Perceptions of Relationship Scale. However, measurement invariance analyses showed noninvariance for the confusion, fatigue, tension, and vigor factors from the Brunel Mood Scale, all of which have invariance of some type in half or more of their items. Mean differences tests (e.g., across time points) are not appropriate when factors display this amount of measurement invariance (Steenkamp & Baumgartner, 1998; Vandenberg & Lance, 2000). Therefore, mean difference tests are not reported for these factors. Analyses also revealed metric noninvariance for the closeness factor from the Perceptions of Relationship Scale. However, research has shown that metric noninvariance has a negligible effect on the results of mean difference tests for latent factors. We thus report the mean difference test of the closeness factor from T2 to T4. See Section S3 for full measurement invariance analyses for all variables to which mean difference tests were applied.

Finally, optional open questions were included on participants' motivations for taking part in the event and their thoughts and feelings as they approached it (T1), whether they would take part in it again, and why (T4), and whether they would recommend it to friends, and why (T4). Responses to these open items are partially reported below. The complete deidentified data set is available at https://github.com/Social-Body-Lab/TTCResearch. To control for clustering (individuals within teams), participants were also asked to provide team identifiers, and these were cross‐referenced with event organizers' records.

## ANALYSES

Mood, perception of relationship, wellbeing, and bonding changes over time were tested using multilevel models, a form of linear regression that accounts for hierarchical data grouping structures. In this study, survey responses across time points (T1–T4) are nested within participants, and participants are nested within teams. The multilevel models used in these analyses had an intercept‐only level‐two random effect of participant nested within an intercept‐only level‐three random effect of team. More complex random effects structures failed to converge. The main predictor was a binary time variable (e.g., T2 to T3).

When a measure other than time was used as a predictor (i.e., when measures were not repeated), a multilevel model with an intercept‐only level‐two random effect of team was used. Again, more complex random effects structures failed to converge. The same procedures were used for models including the interaction of two measures.

All models included participant sex as a covariate—any additional, model‐specific covariates are noted below. All multilevel models were estimated using the *lme4* (Bates et al., 2015) and *lmerTest* (Kuznetsova et al., 2017) packages in R (version 3.5.3; R Core Team, 2020). Multilevel models were chosen for analyses, as the currently available structural equation modeling (SEM) packages for R do not allow for multilevel SEM with data that are nested at three levels (in this case, repeated measures nested within participants who are nested within teams).

To reduce potential measurement error, all factors were made of factor loadings above 0.4—items with factor loadings below 0.4 were excluded (Matsunaga, 2010). Internal consistency and measurement invariance were also checked, and all factors were amended to meet the requirements of the statistical analyses reported below (see above and Section S2 for Cronbach's α and McDonald's omega scores and data reduction procedures for all factors and components, and Section S3 for measurement invariance analyses).

Mediation analyses were conducted using the *mediation* package in R (Tingley et al., 2014). Mediation models consisted of constituent multilevel models that followed the procedures described above.

## RESULTS

**FIGURE 1:**
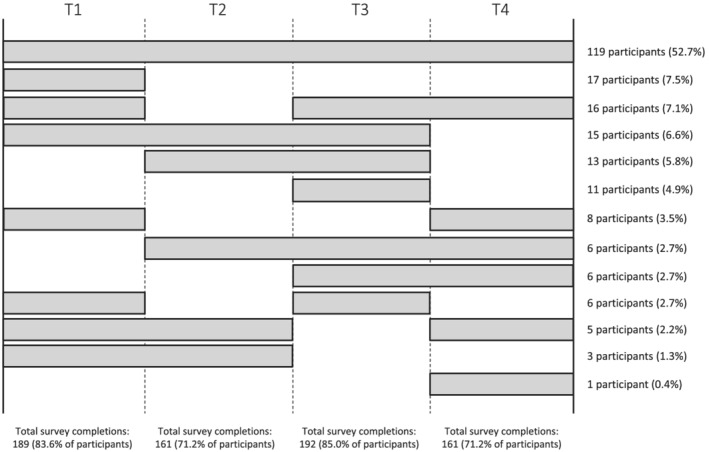
Survey completion chart showing proportions of participants who completed each survey (T1–T4)

Participants were from 102 teams. The mean number of participants from a single team was 2.22 (*SD* = 1.43), with a range of 1–6 and mode of 1. There were 189 completed questionnaires (representing 97 teams) at T1, 161 questionnaires at T2 (representing 74 teams), 192 questionnaires at T3 (representing 85 teams), and 161 questionnaires at T4 (representing 83 teams)—see Figure 1 for details. Of those participants who completed questionnaires at T3 or T4 (*n* = 206), seven withdrew during the challenge (five due to injury, two for unspecified reasons). Two participants who indicated that they had dropped out at T3 did not complete the T4 questionnaire. No participants withdrew from the research. Missing data were handled via listwise deletion.

### Descriptive results

Descriptive data for all measures at T1–T4 are provided in Table 1. Regarding stress appraisal, participants approached the event more as a challenge and less as a threat, suggesting their pre‐event focus was primarily on potentially positive rather than negative experiences and outcomes. Ratings of effort were very high, however, demonstrating that the event was genuinely challenging. Mood before and after the event was generally positive across subscales (see Section S4 for full results). In light of the observed euphoria on the finish line, increases in depression and anger are difficult to interpret but are potentially consistent with the high levels of exhaustion and discomfort that many participants may have been feeling when they were asked to complete the questionnaire. Team relationships were rated highly across subscales of closeness, similarity, and everyday centrality. However, both closeness and everyday centrality decreased from T2 to T4 (both *p* < .001; see Section S4 for full results), although similarity showed no significant change. This suggests that team relationships and engagement were relatively intense going into the event but that they abated soon afterward.

The vast majority of participants confirmed that they would participate in the TTC again (84.47% of T4 questionnaire respondents). Of those who responded “no,” only four gave clearly negative reasons (e.g., “it nearly f***ing killed me”); the majority were satisfied with their accomplishments (e.g., having already completed the challenge across all three distance categories) or wished to focus on other priorities. Reflections in the open questions were mostly positive and focused on sense of achievement, self‐belief and self‐confidence, being and working together as a team, sense of reward (particularly at the finish line), physical fitness, personal growth, atmosphere, fun, friendships made, and skills learned, all against a backdrop of intense challenge.

### Tests of hypotheses

**FIGURE 2:**
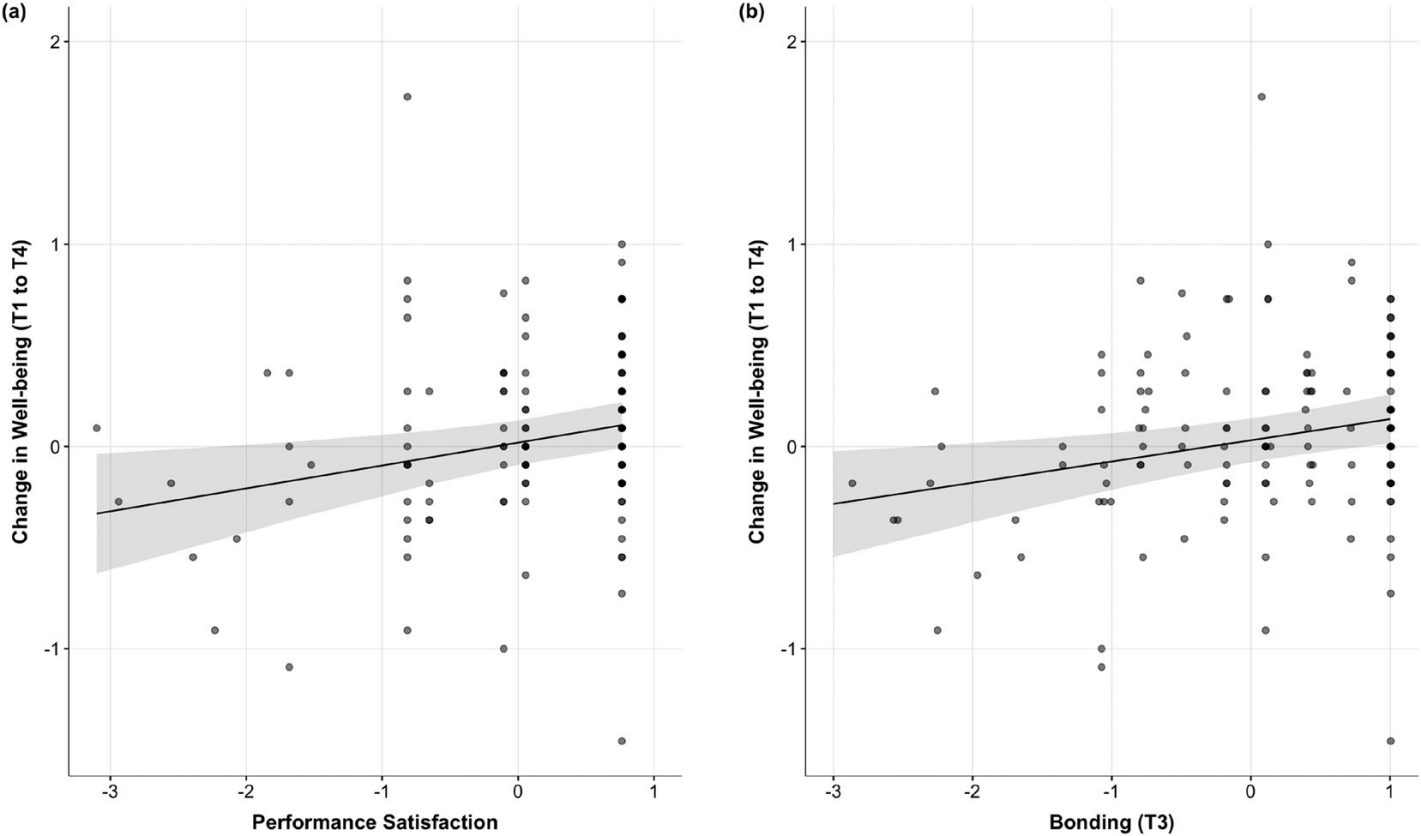
Plot of estimated effects (with shaded 95% confidence interval [CI]) of (a) performance satisfaction (T3) and (b) bonding (T3) on changes in wellbeing (T1–T4), controlling for participant sex

H1: There was a significant increase in wellbeing from T1 to T4 (*b* = 0.079, *SE* = 0.034, *t*[166.2] = 2.330, *p* = .021), as well as a significant effect of sex, with males showing higher wellbeing overall (*b* = 0.223, *SE* = 0.067, *t*[181.6] = 3.35, *p* = .001; see Section S4 for model results).
H2: As shown in Figure 2, the wellbeing increase was significantly predicted by performance satisfaction at T3 (*b* = 0.113, *SE* = 0.041, *t*[136.0] = 2.793, *p* = .006) and bonding at T3 (*b* = 0.107, *SE* = 0.038, *t*[136.0] = 2.808, *p* = .006). Analyses revealed a significant increase in bonding from T2 to T3 (*b* = 0.394, *SE* = 0.056, *t*[159.4] = 7.001, *p* < .001), and a significant decrease from T2 to T4 (*b* = −0.212, *SE* = 0.064, *t*[134.5] = −3.305, *p* = .001). Although trending in the hypothesized direction, change in bonding (T2 amd T3) did not significantly predict the wellbeing increase (T1–T4; *b* = 0.090, *SE* = 0.049, *t*[119.0] = 1.829, *p* = .070), nor did bonding at T2 (*b* = 0.006, *SE* = 0.041, *t*[122.0] = 0.228, *p* = .820). See Section S4 for all model results.
H3: Regarding hypothesized predictors of bonding immediately after the event (T3), analyses revealed a significant positive effect of physical discomfort (*b* = 0.240, *SE* = 0.071, *t*[183.4] = 3.405, *p* < .001), received emotional support (*b* = 0.745, *SE* = 0.071, *t*[190.3] = 10.496, *p* < .001), received esteem support (*b* = 0.688, *SE* = 0.073, *t*[193.0] = 9.47, *p* < .001), experienced interdependence (*b* = 0.528, *SE* = 0.062, *t*[190.0] = 8.523, *p* < .001), and subjective ratings of team performance relative to expectations, controlling for subjective ratings of individual performance relative to expectations (*b* = 0.097, *SE* = 0.048, *t*[188.0] = 2.020, *p* = .045; all T3). See Section S4 for all model results and Section S5 and Figures S5.1–S5.5 for plots of model estimates.

We further examined whether experienced interdependence and physical discomfort were associated with increased bonding from T2 and T3. Experienced Interdependence positively predicted an increase in bonding from T2 to T3 (*b* = 0.177, *SE* = 0.072, *t*[150.3] = 2.438, *p* = .016). There was no significant relationship between physical discomfort and change in bonding from T2 to T3 (*b* = 0.028, *SE* = 0.073, *t*[149.0] = 0.390, *p* = .697).

H4: To examine whether pre‐event bonding (T2) and perceived support (T2; emotional and esteem) buffer the physical stress of the challenge, we analyzed their effects on physical discomfort (controlling for effort). No significant associations were found (see Section S4 for all model results).

### Exploratory analyses

E1: Regarding links between interdependence and bonding at T2, pre‐event bonding (T2) was significantly predicted by Behavioral Interdependence (*b* = 0.273, *SE* = 0.071, *t*[149.0] = 3.830, *p* < .001), though this result should be interpreted cautiously due to the relatively low internal consistency of the behavioral interdependence subscale (Cronbach's α = .55). Pre‐event bonding was also predicted by perceived emotional support (T2; *b* = 0.620, *SE* = 0.081, *t*[157.6] = 7.696, *p* < .001), and perceived esteem support (T2; *b* = 0.633, *SE* = 0.084, *t* = 7.526, *p* < .001).
E2: To explore the relationship between variables associated with the increase in wellbeing, we analyzed post‐event bonding as a predictor of performance satisfaction; bonding at T3 significantly predicted performance satisfaction (*b* = 0.408, *SE* = 0.661, *t*[193.0] = 6.174, *p* < .001; see Section S5 and Figure S5.6 for a plot of model estimates), in line with previous work suggesting an association between team bonding and performance (Davis et al., 2015).
E3: To explore contingencies among challenge, support, and bonding elements, we analyzed interactions between physical discomfort and (a) experienced interdependence or (b) received support (both emotional and esteem) on bonding outcomes (all T3). Analyses revealed a nonsignificant interaction effect on bonding of physical discomfort and experienced interdependence (*b* = 0.019, *SE* = 0.050, *t*[188.0] = 0.382, *p* = .703), physical discomfort and received emotional support (*b* = −0.110, *SE* = 0.067, *t*[188.0] = −1.640, *p* = .103), and physical discomfort and received esteem support (*b* = −0.081, *SE* = 0.062, *t*[188.0] = −1.298, *p* = .194). See Section S4 for all model results and Section S5 and Figures S5.6 and S5.7 for plots of estimates from models with the physical discomfort and received support interactions (both of which show a trend in the hypothesized direction).
E4: Despite their positive elements, team challenge events can also be perceived as threatening and intimidating, potentially reducing their appeal to those who might stand to benefit most. To understand the importance of social factors in mitigating perceived threat prior to the event, we explored whether collective efficacy mediated the relationship between perceived support (both emotional and esteem) and perceptions of threat (all T2). Results showed a nonsignificant average indirect effect of −.015 (95% confidence interval [CI]: [−0.094, 0.060], *p* = .680), a nonsignificant average direct effect of −.095 (95% CI: [−0.291, 0.090], *p* = .360), and a nonsignificant total effect of −.109 (95% CI: [−0.282, 0.070], *p* = .220) of the mediation model with perceived emotional support as the predictor variable. For the model with perceived esteem support as the predictor variable, there was a nonsignificant average indirect effect of .001 (95% CI: [−0.067, 0.070], *p* = .946), a significant average direct effect of −.246 (95% CI: [−0.443, −0.040], *p* = .012), and a significant total effect of −.244 (95% CI: [−0.423, −0.060], *p* = .010): Collective efficacy did not mediate the relationship between perceived esteem support and threat, but perceived esteem support did have a direct and total effect on threat (see Section S4 for all model results). However, as the self‐report variables in these mediation analyses were measured at the same time (T2), temporal precedence from perceived support to collective efficacy to threat can only be theoretically inferred.

## DISCUSSION

This study investigated links among interdependence, social bonding and mental wellbeing in the context of a highly challenging team event. In line with hypotheses and corroborating a broader literature on the links among interdependent relationships, social bonding and wellbeing, the results suggest that experiences of interdependence among peers in a challenging team event are associated with stronger social bonds and higher wellbeing. There was a significant overall increase in participants' self‐reported wellbeing from T1 (7–10 days before the event) to T4 (7–10 days after the event). The increase in wellbeing was predicted by bonding and performance satisfaction immediately upon completion of the event (T3). Given the importance of group bonding for performance success (Davis et al., 2015), we explored the association between bonding and performance satisfaction (both T3); bonding positively predicted performance satisfaction.

The results further suggest that what matters for wellbeing is that team bonds are strong immediately after the event, irrespective of how strong or weak they were before the event. Although there was an increase in Bonding from T2 (the eve of the event) to T3, this did not significantly predict the increase in wellbeing, nor did bonding at T2. Therefore, bonding after the event, rather than prior bonding or changes in bonding through the course of the event, as well as (partly bonding‐related) perceptions of achievement, are associated with post‐event increases in participant wellbeing. This supports the hypothesized links between social bonding, performance success, and wellbeing in the context of a challenging and interdependent collective activity, with higher post‐event bonding levels predicting the positive change in wellbeing. The results corroborate a growing interdisciplinary literature on the significance of even short‐term or one‐off shared, interdependent experiences in forging and sustaining health‐promoting social bonds (Davis et al., 2015; Tarr et al., 2015, 2018; Tunçgenç & Cohen, 2016).

Analyses further aimed to identify which elements of the event contribute to higher post‐event team bonding. On the basis of the previous theory and evidence, we hypothesized positive effects of experienced interdependence (needing and helping one another), received esteem and emotional support, physical pain and fatigue, and perceptions of team performance relative to expectations (i.e., higher scores indicate stronger positive violation of expectation) on post‐event bonding. Hypotheses were supported, suggesting that mutual reliance on one another for help and support in the context of a fatiguing and painful challenge, and (possibly thereby) doing better as a team than expected, can all contribute to stronger intra‐team bonds. Similar associations were revealed among variables measured immediately before the event—perceived mutual dependence (behavioral interdependence) and support (emotional and esteem) predicted bonding at T2, supporting qualitative observations on the significance of pre‐event team training for team cohesion. Finally, experienced interdependence, but not physical discomfort, was associated with a significant increase in Bonding from T2 to T3. The results suggest that the sense of social connection and bonding that is a core component of mental wellbeing is enhanced via multiple pathways. Causal pathways to bonding, via shared pain, joint activity, and mutual dependence, have previously been investigated in isolation in controlled laboratory and field contexts (Bastian et al., 2014; Davis et al., 2015; Tarr et al., 2018). To our knowledge, this is the first study to combine them in a naturalistic setting and to assess associations with wellbeing.

Although relationships among pain, interdependence, and team bonding are often part of the lore of such team challenge events, the results here offer important quantitative support for their functional integration and for their positive association with participants' wellbeing. To further explore potential interactions among these elements, we examined contingencies between the effects of pain/fatigue and received support on bonding. Results revealed no significant interaction effects of received support (esteem or emotional) and physical discomfort on team bonding, suggesting that fatigue/pain and received support are independently associated with bonding among teammates immediately after the event. The positive relationship between received support and bonding at T3 did not vary significantly as a function of reported physical discomfort (see Figures S5.7 and S5.8). We also find no evidence that the relationship between physical discomfort and bonding was contingent on experienced interdependence. Though these results do not offer further support for the expected contingencies, reported levels of received support were high overall, and this may obscure any hypothesized relationships between physical discomfort and received support on bonding. Further research could explore why and how pain and fatigue are independently associated with increased bonding, given high levels of support. Recent research suggests that sharing painful experiences can strengthen cohesion and cooperation in groups and communities (e.g., Kavanagh et al., 2019).

Finally, we explored the impact of social factors on pre‐event perceptions of the event as threatening, specifically the mediating effect of collective efficacy on perceived support (emotional and esteem). Perceived esteem (but not emotional) support had a negative direct effect on perceptions of threat, and neither model revealed a relationship with collective efficacy. These exploratory results tentatively suggest that *esteem support* by team members and others might be an effective target for interventions aimed at encouraging participation among those who view such challenges as more likely to cause them harm than to enable personal growth. Future research examining associations among personality, perceived support, and stress appraisal could shed further light on individual differences contributing to these patterns (Chai & Low, 2015).

We also explored the buffering effects of pre‐event bonding and support on physical discomfort experienced during the event. Physical discomfort was not predicted by pre‐event bonding or perceived emotional or perceived esteem support (controlling for effort); hence, contrary to previous findings in other contexts, we do not find any evidence that team bonds or perceived support ahead of the event buffer the physical stress experienced during it (Davis et al., 2015). Although effort levels were high overall (9.22 out of 10, *SD* = 1.00), pain and fatigue had a comparatively low combined mean score of 6.51 (*SD* = 1.92)—it may be that buffering effects are stronger at greater intensities of pain and fatigue.

The correlational, observation‐based design of this study limits causal inference. However, the hypotheses tested are drawn from existing literature, including controlled experimental designs and evidence supporting causal relationships among challenge/pain, group bonding, social support and interdependence, and wellbeing (Bastian et al., 2014; Cohen et al., 2010; Davis et al., 2015; Davis & Cohen, 2018). Field‐based research in the context of controlled, team‐based events can offer important, complementary evidence from real‐life settings, providing greater ecological validity.

Another limitation is the reliance on self‐report measures. Future research could supplement self‐reported data with other forms of behavioral observation (e.g., physiological effort, prosocial behavior, and objective performance data). More generally, as bonding was already high at T1 (7–10 days prior to the event), further research is needed to investigate whether similar wellbeing effects occur when participants are not highly bonded prior to such events (i.e., in one‐off challenges with little prior team engagement).

Our sample also had a relatively high wellbeing score at T1, suggesting positive mental wellbeing among our participants overall in the run‐up to the event. Although the TTC experience potentially has a positive impact on flourishing (i.e., the “thriving” aspect of wellbeing), it remains to be seen whether such experiences can protect against or alleviate mental illness. Further research is required to evaluate effects of peer‐based mutual reliance in team challenge events among youth with relatively low mental wellbeing or who are specifically vulnerable to mental health problems. Results could complement related research on the utility of group‐based interventions that forge social identifications to protect against and alleviate loneliness (e.g., GROUPS 4 HEALTH; see Haslam et al., 2019), nature based “social prescribing” and therapy (e.g., Leavell et al., 2019), and social connectedness or school belonging interventions that explicitly target and facilitate the development of positive relationships and improve resilience (Allen et al., 2021). A longer term study could also assess the duration and stability of effects on wellbeing.

Finally, scale reliability was suboptimal for our measures of behavioral interdependence and for the mood dimension of anger (T2 only). The measure of anger was taken primarily for descriptive purposes and does not affect our main analyses. The measure of behavioral interdependence was used to test an association between perceived mutual dependence and bonding before the event. The identified positive association should therefore be treated with caution pending follow‐up research with more suitable measures of interdependence (e.g., Gerpott et al., 2018).

## CONCLUSION

This research contributes evidence on psychological pathways linking interdependence, support, bonding and wellbeing in an outdoor team challenge. Results underline the value of organized, challenging, and purposeful youth activities that encourage social bonds, mutual reliance, and collective achievement.

## CONFLICT OF INTEREST

The authors declare that they have no known competing financial interests or personal relationships that could have appeared to influence the work reported in this manuscript.

## ETHICS STATEMENT

The research was approved by the Departmental Research Ethics Committee of the School of Anthropology and Museum Ethnography, University of Oxford, and informed consent was obtained from participants and parents prior to participation.

## Supporting information


**Data S1.** Supporting information

## Data Availability

Anonymized data and R code for analyses are available online (https://github.com/Social-Body-Lab/TTCResearch).

## ENDNOTES

^1^

https://www.gov.uk/government/publications/relationships-education-relationships-and-sex-education-rse-and-health-education

^2^
Due to an error, T3 printed questionnaires did not include measures of pain, fatigue, effort, and actual interdependence (help and need). These questionnaires represent four out of 193 respondents at T3 (3.63%). A total of seven participants at T3 reported having withdrawn during the challenge.

## References

[aphw12351-bib-0001] Abadi, E. , & Gill, D. L. (2020). The role of socializing agents on dropout and continuing participation of adolescent girls in masculine‐typed sports. International Journal of Kinesiology in Higher Education, 4(3), 77–90. 10.1080/24711616.2019.1656118

[aphw12351-bib-0002] Aktipis, A. , Cronk, L. , Alcock, J. , Ayers, J. D. , Baciu, C. , Balliet, D. , Boddy, A. M. , Curry, O. S. , Krems, J. A. , & Muñoz, A. (2018). Understanding cooperation through fitness interdependence. Nature Human Behaviour, 2(7), 429–431. 10.1038/s41562-018-0378-4 31097813

[aphw12351-bib-0003] Allen, K.‐A. , Jamshidi, N. , Berger, E. , Reupert, A. , Wurf, G. , & May, F. (2021). Impact of school‐based interventions for building school belonging in adolescence: A systematic review. Educational Psychology Review, 1–29.34226808

[aphw12351-bib-0004] Balliet, D. , Tybur, J. M. , & Lange, P. A. M. V. (2016). Functional interdependence theory: An evolutionary account of social situations. Personality and Social Psychology Review, 21(4), 361–388. 10.1177/1088868316657965 27466269

[aphw12351-bib-0005] Bastian, B. , Jetten, J. , & Ferris, L. J. (2014). Pain as social glue: Shared pain increases cooperation. Psychological Science, 25(11), 2079–2085. 25193943 10.1177/0956797614545886

[aphw12351-bib-0006] Bates, D. , Mächler, M. , Bolker, B. , & Walker, S. (2015). Fitting linear mixed‐effects models using lme4. Journal of Statistical Software, 67(1), 1–48.

[aphw12351-bib-0007] Baumeister, R. F. , & Leary, M. R. (1995). The need to belong: Desire for interpersonal attachments as a fundamental human motivation. Psychological Bulletin, 117(3), 497–529. 7777651

[aphw12351-bib-0008] Beckes, L. , & Coan, J. A. (2011). Social baseline theory: The role of social proximity in emotion and economy of action. Social and Personality Psychology Compass, 5(12), 976–988. 10.1111/j.1751-9004.2011.00400.x

[aphw12351-bib-0009] Butler, J. , & Kern, M. L. (2016). The PERMA‐profiler: A brief multidimensional measure of flourishing. International Journal of Wellbeing, 6(3), 1–48. 10.5502/ijw.v6i3.526

[aphw12351-bib-0010] Cacioppo, J. T. , & Cacioppo, S. (2018). The growing problem of loneliness. The Lancet, 391(10119), 426. 10.1016/s0140-6736(18)30142-9 PMC6530780 29407030

[aphw12351-bib-0011] Castro, V. F. , & Pacherie, E. (2020). Joint actions, commitments and the need to belong. Synthese, 1‐30. 10.1007/s11229-020-02535-0

[aphw12351-bib-0012] Chai, M. S. , & Low, C. S. (2015). Personality, coping and stress among university students. American Journal of Applied Psychology, 4(3–1), 33–38.

[aphw12351-bib-0013] Clarke, A. , Friede, T. , Putz, R. , Ashdown, J. , Martin, S. , Blake, A. , Adi, Y. , Parkinson, J. , Flynn, P. , Platt, S. , & Stewart‐Brown, S. (2011). Warwick‐Edinburgh Mental Well‐being Scale (WEMWBS): Validated for teenage school students in England and Scotland. A mixed methods assessment. BMC Public Health, 11(1). 10.1186/1471-2458-11-487 PMC3141456 21693055

[aphw12351-bib-0014] Coan, J. A. , & Sbarra, D. A. (2015). Social baseline theory: The social regulation of risk and effort. Current Opinion in Psychology, 1, 87–91. 10.1016/j.copsyc.2014.12.021 25825706 PMC4375548

[aphw12351-bib-0015] Cohen, E. , Ejsmond‐Frey, R. , Knight, N. , & Dunbar, R. I. (2010). Rowers' high: Behavioural synchrony is correlated with elevated pain thresholds. Biology Letters, 6(1), 106–108. 19755532 10.1098/rsbl.2009.0670 PMC2817271

[aphw12351-bib-0016] Cohen, S. (2004). Social relationships and health. American Psychologist, 59(8), 676–684. 10.1037/0003-066x.59.8.676. PMID ‐ 15554821. 15554821

[aphw12351-bib-0017] Cohen, S. , & Wills, T. A. (1985). Stress, social support, and the buffering hypothesis. Psychological Bulletin, 98(2), 310–357. 10.1037/0033-2909.98.2.310 3901065

[aphw12351-bib-0018] Cruwys, T. , Haslam, S. A. , Dingle, G. A. , Haslam, C. , & Jetten, J. (2014). Depression and social identity: An integrative review. Personality and Social Psychology Review, 18(3), 215–238. 24727974 10.1177/1088868314523839

[aphw12351-bib-0019] Davis, A. , & Cohen, E. (2018). The effects of social support on strenuous physical exercise. Adaptive Human Behavior and Physiology, 4(2), 171–187. 29755928 10.1007/s40750-017-0086-8 PMC5935032

[aphw12351-bib-0020] Davis, A. , Taylor, J. , & Cohen, E. (2015). Social bonds and exercise: Evidence for a reciprocal relationship. PLoS ONE, 10(8), e0136705. 10.1371/journal.pone.0136705.s012 26317514 PMC4552681

[aphw12351-bib-0021] Davis, A. J. , Crittenden, B. , & Cohen, E. (2021). Effects of social support on performance outputs and perceived difficulty during physical exercise. Physiology and Behavior, 113490. 34139269 10.1016/j.physbeh.2021.113490

[aphw12351-bib-0022] Davis, A. J. , MacCarron, P. , & Cohen, E. (2021). Social reward and support effects on exercise experiences and performance: Evidence from parkrun. PLoS ONE, 16(9), e0256546. 34525097 10.1371/journal.pone.0256546 PMC8443045

[aphw12351-bib-0023] Dunbar, R. I. (2018). The anatomy of friendship. Trends in Cognitive Sciences, 22(1), 32–51. 29273112 10.1016/j.tics.2017.10.004

[aphw12351-bib-0024] Dunbar, R. I. M. , & Shultz, S. (2010). Bondedness and sociality. Behaviour, 147(7), 775–803.

[aphw12351-bib-0025] Freeman, P. , Coffee, P. , Moll, T. , Rees, T. , & Sammy, N. (2014). The ARSQ: The Athletes' Received Support Questionnaire. Journal of Sport and Exercise Psychology, 36(2), 189–202. 24686955 10.1123/jsep.2013-0080

[aphw12351-bib-0026] Freeman, P. , Coffee, P. , & Rees, T. (2011). The PASS‐Q: The perceived available support in sport questionnaire. Journal of Sport and Exercise Psychology, 33(1), 54–74. 21451171 10.1123/jsep.33.1.54

[aphw12351-bib-0027] Gerpott, F. H. , Balliet, D. , Columbus, S. , Molho, C. , & de Vries, R. E. (2018). How do people think about interdependence? A multidimensional model of subjective outcome interdependence. Journal of Personality and Social Psychology, 115(4), 716. 28872331 10.1037/pspp0000166

[aphw12351-bib-0028] Gunnell, D. , Kidger, J. , & Elvidge, H. (2018). Adolescent mental health in crisis. British Medical Journal, 361, k2608. 29921659 10.1136/bmj.k2608

[aphw12351-bib-0029] Haslam, C. , Cruwys, T. , Chang, M. X.‐L. , Bentley, S. V. , Haslam, S. A. , Dingle, G. A. , & Jetten, J. (2019). GROUPS 4 HEALTH reduces loneliness and social anxiety in adults with psychological distress: Findings from a randomized controlled trial. Journal of Consulting and Clinical Psychology, 87(9), 787. 31403815 10.1037/ccp0000427

[aphw12351-bib-0030] Hawkley, L. C. , & Cacioppo, J. T. (2010). Loneliness matters: A theoretical and empirical review of consequences and mechanisms. Annals of Behavioral Medicine, 40(2), 218–227. 10.1007/s12160-010-9210-8 20652462 PMC3874845

[aphw12351-bib-0031] Henry, K. B. , Arrow, H. , & Carini, B. (1999). A tripartite model of group identification theory and measurement. Small Group Research, 30(5), 558–581. 10.1177/104649649903000504

[aphw12351-bib-0032] Holt‐Lunstad, J. , & Uchino, B. (2008). Social support and health. In K. Glanz , B. K. Rimer , & K. Viswanath (Eds.), Health behavior and health education: Theory, research, and practice. John Wiley & Sons.

[aphw12351-bib-0033] Hornstein, E. A. , Fanselow, M. S. , & Eisenberger, N. I. (2016). A safe haven: Investigating social‐support figures as prepared safety stimuli. Psychological Science, 27(8), 1051–1060. 10.1177/0956797616646580 27324266

[aphw12351-bib-0034] Jetten, J. , Haslam, C. , & Alexander, S. H. (2012). The social cure: Identity, health and well‐being. Psychology Press.

[aphw12351-bib-0035] Kavanagh, C. M. , Jong, J. , McKay, R. , & Whitehouse, H. (2019). Positive experiences of high arousal martial arts rituals are linked to identity fusion and costly pro‐group actions. European Journal of Social Psychology, 49(3), 461–481. 31598015 10.1002/ejsp.2514 PMC6774318

[aphw12351-bib-0036] Kuznetsova, A. , Brockhoff, P. , & RHB, C. (2017). Lmertest package: Tests in linear mixed effects models. Journal of Statistical Software, 82(13), 1026. 10.18637/jss.v082.i13

[aphw12351-bib-0037] Leavell, M. , Leiferman, J. , Gascon, M. , Braddick, F. , Gonzalez, J. , & Litt, J. (2019). Nature‐based social prescribing in urban settings to improve social connectedness and mental well‐being: A review. Current Environmental Health Reports, 6(4), 297–308. 31713144 10.1007/s40572-019-00251-7

[aphw12351-bib-0038] Lennon, M. (2021). The state of children's mental health services 2020/21. https://www.childrenscommissioner.gov.uk/wp-content/uploads/2021/01/cco-the-state-of-childrens-mental-health-services-2020-21-tech-report.pdf

[aphw12351-bib-0039] Loades, M. E. , Chatburn, E. , Higson‐Sweeney, N. , Reynolds, S. , Shafran, R. , Brigden, A. , Linney, C. , McManus, M. N. , Borwick, C. , & Crawley, E. (2020). Rapid systematic review: The impact of social isolation and loneliness on the mental health of children and adolescents in the context of COVID‐19. Journal of the American Academy of Child and Adolescent Psychiatry, 59(11), 1218–1239.e1213. 10.1016/j.jaac.2020.05.009 32504808 PMC7267797

[aphw12351-bib-0040] Matsunaga, M. (2010). How to factor‐analyze your data right: Do's, don'ts, and how‐to's. International Journal of Psychological Research, 3(1), 97–110.

[aphw12351-bib-0041] Michael, J. , Sebanz, N. , & Knoblich, G. (2016). Observing joint action: Coordination creates commitment. Cognition, 157(C), 106–113. 10.1016/j.cognition.2016.08.024 27610745 PMC5146639

[aphw12351-bib-0042] Páez, D. , Rimé, B. , Basabe, N. , Wlodarczyk, A. , & Zumeta, L. (2015). Psychosocial effects of perceived emotional synchrony in collective gatherings. Journal of Personality and Social Psychology, 108(5), 711–729. 10.1037/pspi0000014 25822033

[aphw12351-bib-0043] Peacock, E. J. , & Wong, P. T. P. (1990). The Stress Appraisal Measure (SAM): A multidimensional approach to cognitive appraisal. Stress Medicine, 6, 227–236. 10.2307/40729741?ref=search-gateway:cebc147346729d5ce5e1fe9ebc09fd2f

[aphw12351-bib-0044] R Core Team . (2020). R: A language and environment for statistical computing. R foundation for computing. Vienna, Austria.

[aphw12351-bib-0045] Rowland, L. , & Curry, O. S. (2019). A range of kindness activities boost happiness. The Journal of Social Psychology, 159(3), 340–343. 29702043 10.1080/00224545.2018.1469461

[aphw12351-bib-0046] Sbarra, D. A. , & Coan, J. A. (2018). Relationships and health: The critical role of affective science. Emotion Review, 10(1), 40–54. 10.1177/1754073917696584

[aphw12351-bib-0047] Singh, P. , Tewari, S. , Kesberg, R. , Karl, J. A. , Bulbulia, J. , & Fischer, R. (2020). Time investments in rituals are associated with social bonding, affect and subjective health: A longitudinal study of Diwali in two Indian communities. Philosophical Transactions of the Royal Society B: Biological Sciences, 375(1805), 20190430. 10.1098/rstb.2019.0430. PMID ‐ 32594880. PMC7423265 32594880

[aphw12351-bib-0048] Slavich, G. M. (2020). Social safety theory: A biologically based evolutionary perspective on life stress, health, and behavior. Annual Review of Clinical Psychology, 16. 10.1146/annurev-clinpsy-032816-045159 PMC7213777 32141764

[aphw12351-bib-0049] Stanger, N. , Backhouse, S. H. , Jennings, A. , & McKenna, J. (2018). Linking motivational climate with moral behavior in youth sport: The role of social support, perspective taking, and moral disengagement. Sport, Exercise, and Performance Psychology, 7(4), 392–407. 10.1037/spy0000122

[aphw12351-bib-0050] Steenkamp, J.‐B. E. , & Baumgartner, H. (1998). Assessing measurement invariance in cross‐national consumer research. Journal of Consumer Research, 25(1), 78–90.

[aphw12351-bib-0051] Swann, W. B. , Gomez, A. , Seyle, D. C. , Morales, J. , & Huici, C. (2009). Identity fusion: The interplay of personal and social identities in extreme group behavior. Journal of Personality and Social Psychology, 96(5). 10.1037/a0013668 19379032

[aphw12351-bib-0052] Tarr, B. , Launay, J. , Cohen, E. , & Dunbar, R. (2015). Synchrony and exertion during dance independently raise pain threshold and encourage social bonding. Biology Letters, 11(10), 20150767. 10.1098/rsbl.2015.0767 26510676 PMC4650190

[aphw12351-bib-0053] Tarr, B. , Slater, M. , & Cohen, E. (2018). Synchrony and social connection in immersive virtual reality. Scientific Reports, 8(1), 3693. 10.1038/s41598-018-21765-4 29487405 PMC5829252

[aphw12351-bib-0054] Tennant, R. , Hiller, L. , Fishwick, R. , Platt, S. , Joseph, S. , Weich, S. , Parkinson, J. , Secker, J. , & Stewart‐Brown, S. (2007). The Warwick‐Edinburgh Mental Well‐being Scale (WEMWBS): Development and UK validation. Health and Quality of Life Outcomes, 5, 63. 18042300 10.1186/1477-7525-5-63 PMC2222612

[aphw12351-bib-0055] Terry, P. C. , Lane, A. M. , Lane, H. J. , & Keohane, L. (1999). Development and validation of a mood measure for adolescents. Journal of Sports Sciences, 17(11), 861–872. 10585166 10.1080/026404199365425

[aphw12351-bib-0056] Tingley, D. , Yamamoto, T. , Hirose, K. , Keele, L. , & Imai, K. (2014). Mediation: R package for causal mediation analysis. Journal of Statistical Software, 59(5), 1–38. 10.18637/jss.v059.i05 26917999

[aphw12351-bib-0057] Tunçgenç, B. , & Cohen, E. (2016). Interpersonal movement synchrony facilitates pro‐social behavior in children's peer‐play. Developmental Science, 21(1), e12505. 10.1111/desc.12505 27990719

[aphw12351-bib-0058] Uchino, B. N. , Cacioppo, J. T. , & Kiecolt‐Glaser, J. K. (1996). The relationship between social support and physiological processes: A review with emphasis on underlying mechanisms and implications for health. Psychological Bulletin, 119(3), 488. 8668748 10.1037/0033-2909.119.3.488

[aphw12351-bib-0059] Vandenberg, R. J. , & Lance, C. E. (2000). A review and synthesis of the measurement invariance literature: Suggestions, practices, and recommendations for organizational research. Organizational Research Methods, 3(1), 4–70.

[aphw12351-bib-0060] Vangelisti, A. L. , & Caughlin, J. P. (1997). Revealing family secrets: The influence of topic, function, and relationships. Journal of Social and Personal Relationships, 14(5), 679–705. 10.1177/1077800406294947

[aphw12351-bib-0061] Williams, R. , & Scott, C. (2019). The current state of outdoor learning in a UK secondary setting: Exploring the benefits, drawbacks and recommendations. ABC Journal of Advanced Research, 8(2), 109–122.

[aphw12351-bib-0062] Wolf, W. , Launay, J. , & Dunbar, R. I. (2016). Joint attention, shared goals, and social bonding. British Journal of Psychology, 107(2), 322–337. 26256821 10.1111/bjop.12144 PMC4849556

[aphw12351-bib-0063] World Health Organization . (2020). Adolescent mental health [fact sheet]. Retrieved October 15, from https://www.who.int/news-room/fact-sheets/detail/adolescent-mental-health

[aphw12351-bib-0064] Zumeta, L. N. , Oriol, X. , Telletxea, S. , Amutio, A. , & Basabe, N. (2016). Collective efficacy in sports and physical activities: Perceived emotional synchrony and shared flow. Frontiers in Psychology, 6(1572), 720. 10.1086/678698 PMC4700277 26779077

