# Supplementary material for: Interdependence, bonding and support are associated with improved mental wellbeing following an outdoor team challenge
Source: Appl Psychol Health Well Being. 2022 Feb 28;15(1):193–216. doi: 10.1111/aphw.12351 (PMC10078634; doi:10.1111/aphw.12351)
Supplement: Supplementary file 1 — Data S1. Supporting information [file APHW-15-193-s001.pdf]

Interdependence, bonding and support are associated with improved mental well-being following an outdoor team challenge

Supplementary Online Material

1 Ten Tors Challenge 2017

The 2017 TTC was held from May 5 – 7, 2017. For further general information on the TTC, see https://www.tentors.org.uk/. Images from the research are included below.

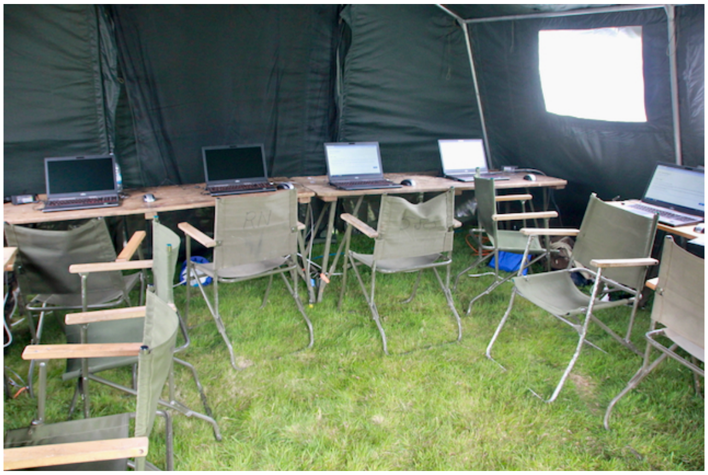

Plate S1.1 The ‘lab tent’ with internet-enabled computer terminals.

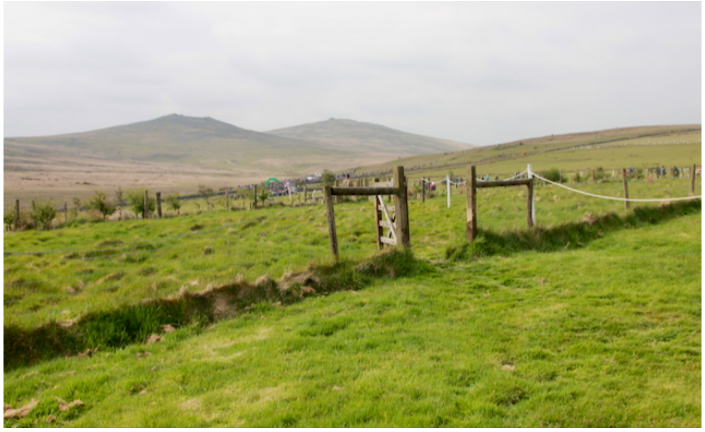

Plate S1.2 Outlook from the lab tent to the Dartmoor terrain. A bright green arch (just visible) indicates the finish line, after which teams are corralled toward the lab tent and beyond to the exit and camp.

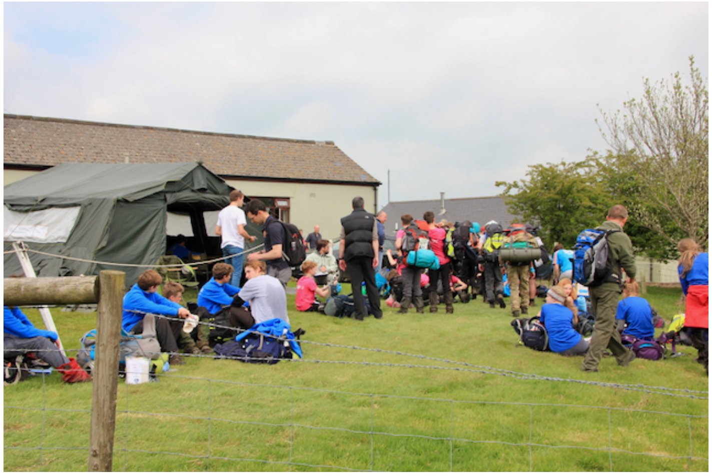

Plate S1.3 Teams gathered and reunited with adult team coaches at the lab tent after completing the Challenge (T3).

2 Confirmatory factor and principal component analyses

This section reports the confirmatory factor and principal component analyses used to confirm previously validated factors and reduce variables measured by multiple items into single components. Variable names provided in parentheses and in single quotes represent variables in the datasets and R scripts used for analyses (datasets and R scripts are available at https://github.com/Social-Body-Lab/TTCResearch). Confirmatory factor and principal component analyses were carried out using R's *lavaan* (Rosseel, 2012) and *psych* (Revelle & Revelle, 2015) packages, respectively.

2.1 Confirmatory factor analyses

2.1.1 Stress Appraisal Measure (T2) – Threat Outcome, Threat Emotion, Challenge, and Centrality

A confirmatory factor analysis (CFA) was carried out on the twelve questions making up the Stress Appraisal Measure (Peacock & Wong, 1990). Three models were compared – a zero-factor model, a three-factor model (Peacock & Wong, 1990) comprised of threat, challenge, and centrality factors, and a four-factor model, which retained the challenge and centrality factors, but split the threat factor into a factor comprised of the two questions on the negative impact of the event ("the event will have a negative impact on you" and "the outcome will be negative") and a factor comprised of the questions related to threat and anxiety ("it's a threatening situation" and it makes them "anxious"). The four factor model was motivated by the result of the three factor model: the threat factor in the three factor model had low factor loadings (the questions about a threatening situation and anxiety had factor loadings below .4, which are considered weak) and the correlation matrix of these four questions suggested that the threat factor be split into two. The four factor model fit significantly better than both the zero factor model, χ^2 (16) = 357.33, p < .001, and the three factor model, χ^2 (4) = 18.39, p = .001. The four factor model had a comparative fit index (CFI) of .79. Factor loadings, Cronbach's α, and McDonald's ω values for each factor can be found in Table S2.1 – factor loadings for all items were above the recommended minimum of 0.4

Table S2.1 Factor loadings and Cronbach's α and McDonald's ω values for the four factor model of the Stress Appraisal Measure at T2

**Factor**	**Factor loading**	**α, ω**
Threat Emotion ("Please rate the extent to which you think/feel …")		.67*
"… it is a threatening situation"	.83	
"… anxious"	.63	
Threat Outcome ("Please rate the extent to which you think/feel …")		.78*
"… the outcome will be negative"	.84	
"... it will have a negative impact on you"	.76	
Challenge ("Please rate the extent to which you think/feel …")		.71, .74
"… it will have a positive impact on you"	.55	
"… you are eager to tackle it"	.65	
"… you can become a stronger person through it"	.57	
"… you are excited about the outcome"	.72	
Centrality ("Please rate the extent to which you think/feel …")		.70, .74
"… it will have important consequences for you"	.67	
"… you will be affected by it"	.57	
"… it will have serious implications for you"	.53	
"… it will have long-term consequences for you"	.68	

* McDonald's ω cannot be calculated for two item factors.

(Matsunaga, 2010). Table S2.1 shows the factor loadings for the preferred four factor model; all factor loadings were above the minimum threshold of 0.4 set by previous research (Matsunaga, 2010).

Finally, a principal component analysis (PCA) was used to extract two components from the four questions on threat. One component, Threat Outcome ('threat_t2_outcome'), was comprised of the two questions on the negative impact of the event and the other component, Threat Emotion ('threat_t2_emotion'), was comprised of the questions related to threat and anxiety. The Threat Outcome component explained 82.08% of the variance in the two questions and had a Cronbach's α of .78, while the Threat Emotion component explained 75.89% of the variance in the two questions and had a Cronbach's α of .67 (McDonald's ω cannot be calculated for two item factors).

The challenge ('challenge_t2') and centrality ('centrality_t2') variables were created by taking the average of the four sub questions that comprised the factors (Peacock & Wong, 1990).

2.1.2 Collective Efficacy (T2)

A CFA was run on the four questions making up the Collective Efficacy for Sport Scale. The one factor model proposed (Zumeta, Oriol, Telletxea, Amutio, & Basabe, 2016) was compared to a null model (no factors). The one factor model fit significantly better than the null model, χ^2 (5) = 214.4, p = .001, had a CFI of .73, and, as shown in Table S2.2, the factor loadings for all items were above the recommended minimum of 0.4 (Matsunaga, 2010).

Table S2.2 Factor loadings and Cronbach's α and McDonald's ω values for the four factor model of Collective Efficacy in sports at T2

**Factor**	**Factor loading**	**α, ω**
Collective efficacy ("Do you think your team …")		.79, .92
"… has more abilities than other teams?"	.67	
"… is effectively prepared for the activities?"	.57	
"… has the ability to overcome distractions?"	.53	
"… can perform the activity better than the other teams?"	.68	

The variables for this factor ('collective_efficacy_t2') was created by averaging responses to all four questions (Zumeta et al., 2016). Cronbach's α and McDonald's ω values indicated good internal consistency for this factor (α = .79, ω = 92).

2.1.3 Perceived Support (T2) – Perceived Emotional and Esteem Support

A CFA was run on the eight questions making up the Perceived Available Support in Sport Questionnaire (PASS-Q). The two factor model proposed (Freeman, Coffee, & Rees, 2011) – comprised of "emotional" and "esteem" support components – was compared to a one factor model and a null model (no factors). The two factor model fit significantly better than the null model, χ^2 (9) = 847.37, p = .001, but not the one factor model, χ^2 (1) = −182.8, p = 1; it had a CFI of .70. The two factor model was chosen over the one factor model because of precedence in the literature (Freeman et al., 2011). As shown in Table S2.3, the factor loadings for all items were above the recommended minimum of 0.4 (Matsunaga, 2010).

The Perceived Emotional Support ('perceived_support_t2_emotional') and Perceived Esteem Support ('perceived_support_t2_esteem') factors were created by taking the average of the four questions making up each component of the PASS-Q. Cronbach's α and McDonald's ω values indicated good internal consistency for both factors (see Table S2.3).

2.1.4 Received Support (T3) – Received Emotional and Esteem Support

A CFA was run on the ten questions making up the Athlete's Received Support Questionnaire (ARSQ). The two factor model proposed (Freeman, Coffee, Moll, Rees, & Sammy, 2014) – comprised of "emotional" and "esteem" support components – was compared to a one factor model and a null model (no factors). The two factor model fit significantly better than the null model, χ^2 (11) = 1,220.6, p = .001, but not the one factor model, χ^2 (1) = −279.9, p = 1. The one factor model had a CFI of .68. The two factor model was chosen over the one factor model because of precedence in the literature (Freeman et al., 2014). As shown in Table S2.4, all factor loadings for both factors were above the recommended minimum of 0.4 (Matsunaga, 2010).

The Received Emotional Support ('received_support_t3_emotional') and Received Esteem Support ('received_support_t3_esteem') factors were created by taking the average of the five sub-questions making up the Received Emotional Support and Received Esteem Support elements of the ARSQ (see Table S2.4). Cronbach's α and McDonald's ω values indicated good internal consistency for both factors (see Table S2.4).

Table S2.3 Factor loadings and Cronbach's α and McDonald's ω values for the two factor model of Perceived Emotional and Esteem Support at T2

Factor	Factor loading	α, ω
Perceived available support ("If needed, to what extent would someone in your team…")		
Emotional Support component of the PASS-Q		.87, .89
"… provide you with comfort and security?"[1]	.81	
"… always be there for you?"[1]	.78	
"… care for you?"[1]	.75	
"… show concern for you?"[1]	.79	
Esteem Support component of the PASS-Q		.88, .91
"… reinforce the positives?"[2]	.77	
"… enhance your self-esteem?"[2]	.72	
"… instill you with the confidence to deal with pressure?"[2]	.82	
"… boost your sense of competence?"[2]	.88	

2.1.5 Mood (T2) – Tension, Vigor, Confusion, Fatigue, Depression, and Anger

A CFA was run on the 24 items making up the Brunel Mood Scale. The six factor model proposed (Terry, Lane, Lane, & Keohane, 1999) was compared to the null model (no factors). The six factor model fit significantly better than the null model, χ^2 (30) = 1,030.7, *p* = .001, had a CFI of .71, and, as shown in Table S2.5, the factor loadings for all but one of the items ("Angry") was above the recommended minimum of 0.4 (Matsunaga, 2010).

The six factor model proposed was thus confirmed, and 23 of the 24 items loaded highly onto their respective factors (Table S2.6), with the exception of "Angry" sub-question on the Anger factor. The variables for each factor ('tension_t2', 'vigour_t2', 'confusion_t2', 'fatigue_t2', 'depression_t2', 'anger_t2') were created by averaging responses to each of the four sub-questions comprising the factor, except that the "angry" sub-question was excluded from the Anger factor, making it the only of the six factors to be comprised of three instead of four sub-questions (as proposed by Terry et al., 1999). Cronbach's α and McDonald's ω values indicated good internal consistency for all factors except the Anger factor (see Table S2.5).

2.1.6 Mood (T3) – Tension, Vigor, Confusion, Fatigue, Depression, and Anger

A CFA was run on the 24 items making up the Brunel Mood Scale. The six factor model proposed (Terry et al., 1999) was compared to the a null model (no factors). The six factor model fit significantly better than the null model, χ^2 (30) = 1,897.8, *p* = .001, had a CFI of .76, and, as shown in Table S2.5, the factor loadings for each item was above the recommended minimum of 0.4 (Matsunaga, 2010).

Given that the six factor model proposed was confirmed, and that the 24 items loaded highly onto their respective factors, the variables for each factor ('tension_t3', 'vigour_t3', 'confusion_t3', 'fatigue_t3', 'depression_t3', 'anger_t3') were created by averaging responses to each of the four

Table S2.4 Factor loadings and Cronbach's α and McDonald's ω values for the two factor model of Received Emotional and Esteem Support at T3

**Factor**	**Factor loading**	**α, ω**
Received support ("How much did your teammates… ")		
Emotional Support component of the ARSQ		.84, .88
"… cheer you up?"	.77	
"… show concern for you?"	.65	
"… make you feel that they would always be there for you?	.74	
"… comfort you?"	.81	
"… listen to you?"	.65	
Esteem Support component of the ARSQ		.88, .92
"…. emphasize your abilities?"	.61	
"… reinforce the positives?"	.70	
"… tell you, you can do it?	.81	
"… encourage you?"	.91	
"... boost your confidence?"	.88	

sub-questions comprising the factor. Cronbach's α and McDonald's ω values indicated good internal consistency for all six factors (see Table S2.5).

2.1.7 Perceptions of Relationship (T2) – Closeness, Similarity, and Everyday Centrality

A CFA was run on the 15 items making up the Perceptions of Relationship Scale. The three factor model (Vangelisti & Caughlin, 1997) was compared to the a null model (no factors). The three factor model fit significantly better than the null model, χ^2 (18) = 824.7, p = .001, had a CFI of .69, and, as shown in Table S2.6, the factor loadings for all but two items – related to shared beliefs – were above the recommended minimum of 0.4 (Matsunaga, 2010).

The three factor model was thus confirmed, with 13 of the 15 factors loading highly onto their respective items (see Table S2.6). The two sub-questions related to beliefs – "My team members and I have very different values" (reversed scored) and "How important are your team members' opinions to you?" – both had factor loadings below the recommended cut-off of 0.4 (Matsunaga, 2010). They were thus not included in the variables for each factor ('closeness_t2', 'similarity_t2', 'eday_centrality_t2'), which were created by averaging responses to each of the sub-questions comprising the factor. Cronbach's α and McDonald's ω values indicated good internal consistency for all three factors (see Table S2.6).

2.1.8 Perceptions of Relationship (T4) – Closeness, Similarity, and Everyday Centrality

A CFA was run on the 15 items making up the Perceptions of Relationship Scale. The three factor model (Vangelisti & Caughlin, 1997) was compared to the a null model (no factors). The three factor model fit significantly better than the null model, χ^2 (18) = 1,301.2, p = .001, had a CFI of .74, and, as shown in Table S2.6, the factor loadings for all but one item – related to shared values – were above the recommended minimum of 0.4 (Matsunaga, 2010).

Table S2.5 Factor loadings and Cronbach's α and McDonald's ω values for the six factor model of the Brunel Mood Scale at T2 and T3

Factor	Factor loading (T2)	α, ω (T2)	Factor loading (T3)	α, ω (T3)
Tension ("How do you feel right now?")		.89, .91		.81, .89
"Panicky"	.78		.61	
"Anxious"	.80		.83	
"Worried"	.86		.66	
"Nervous"	.81		.78	
Vigor ("How do you feel right now?")		.75, .78		.76, .80
"Lively"	.72		.84	
"Energetic"	.84		.86	
"Active"	.65		.72	
"Alert"	.46		.41	
Confusion ("How do you feel right now?")		.61, .70		.75, .80
"Confused"	.57		.55	
"Mixed-up"	.55		.65	
"Muddled"	.69		.57	
"Uncertain"	.51		.72	
Fatigue ("How do you feel right now?")		.80, .86		.85, .87
"Worn out"	.53		.67	
"Exhausted"	.65		.57	
"Sleepy"	.84		.53	
"Tired"	.81		.68	
Depression ("How do you feel right now?")		.74, 89		.90, .91
"Depressed"	.56		.74	
"Downhearted"	.73		.85	
"Unhappy"	.57		.90	
"Miserable"	.82		.84	
Anger ("How do you feel right now?")		.55, .57*		.81, .83
"Annoyed"	.68		.69	
"Bitter"	.53		.83	
"Angry"	.20		.88	
"Bad tempered"	.43		.83	

* These are the Cronbach's α and McDonald's ω values for the final factor, which was comprised of all sub-questions with factor loadings above 0.4 at both timepoints.

Table S2.6 Factor loadings for the three factor model of Perceptions of Relationship Scale at T2 and T4

**Factor**	**Factor loading (T2)**	**α, ω (T2)**	**Factor loading (T4)**	**α, ω (T4)**
Closeness		.80, .90*		.88, .92*
"How close are you to your team members?"	.76		.78	
"How much do you like your team members?"	.75		.82	
"How often do you talk about personal things with your team members?"	.47		.61	
"How important are your team members' opinions to you?"	.32		.65	
"How satisfied are you with your relationship to your team members?"	.50		.74	
"How much do you enjoy spending time with your team members?"	.83		.83	
"How important is your relationship with your team members?"	.74		.77	
Similarity**		.87, .89*		.92*, –
"My team members and I like a lot of the same things."	.80		.88	
"My team members and I share a lot of the same attitudes about things."	.86		.85	
"My team members and I have very different values."[1]	.32		.19	
"My team members and I have a similar outlook on life."	.82		.83	
"My team members and I are very similar."	.71		.88	
Everyday Centrality		.76, .77		.88, .88
"How often do you see your team members?"	.79		.85	
"How central are your team members to your everyday life?"	.70		.83	
"How often do you talk to your team members?"	.57		.83	

1. Reversed scored

* These are the Cronbach's α and McDonald's ω values for the final factor, which was comprised of all sub-questions with factor loadings above 0.4 at both timepoints.

** The McDonald's ω value for the Similarity factor could not be calculated due to model convergence failure.

The three factor model was thus confirmed, with 14 of the 15 factors loading highly onto their respective items (see Table S2.7). The sub-question related to value – "My team members and I have very different values" (reversed scored) – had factor loadings below the recommended cut-off of 0.4 (Matsunaga, 2010). This sub-question was thus not included in the variables making up the Similarity factor. Variables for each factor ('closeness_t4', 'similarity_t4', 'eday_centrality_t4') were created by averaging responses to each of the sub-questions comprising the factor. Cronbach's α and McDonald's ω values indicated good internal consistency for all three factors (see Table S2.6).

2.1.9 Warwick-Edinburgh Mental Wellbeing Scale (T1)

A CFA was run on the 14 items making up the Warwick-Edinburgh Mental Wellbeing Scale. The one factor model proposed (Terry et al., 1999) was compared to the a null model (no factors). The one factor model fit significantly better than the null model, χ^2 (15) = 582.1, p = .001, had a CFI of .84, and, as shown in Table S2.7, the factor loadings for all but three items were above the recommended minimum of 0.4 (Matsunaga, 2010).

The variable ('wellbeing_t1') for this factor was created by averaging responses to all of the sub-questions with factor loadings above 0.4. The questions on interests ("I've been feeling interested in other people", "I've been interested in new things") and love ("I've been feeling loved") were not included in the eleven sub-questions that made up the final variable due to their factor loadings being below the recommended minimum (Matsunaga, 2010). Cronbach's α and McDonald's ω values indicated good internal consistency (α = .84, ω = .87) for this factor.

2.1.10 Warwick-Edinburgh Mental Wellbeing Scale (T4)

A CFA was run on the 14 items making up the Warwick-Edinburgh Mental Wellbeing Scale. The one factor model proposed (Terry et al., 1999) was compared to the a null model (no factors). The one factor model fit significantly better than the null model, χ^2 (15) = 655.9, p = .001, had a CFI of .78, and, as shown in Table S2.7, the factor loadings for all items were above the recommended minimum of 0.4 (Matsunaga, 2010).

The variable ('wellbeing_t4') for this factor was created by averaging responses to all of the sub-questions in the scale that had a factor loading over 0.4 *and* that were included in the creation of the 'wellbeing_t1' variable (see 2.1.9). Thus, although all items in the Warwick-Edinburgh Mental Wellbeing Scale had factor loadings about 0.4 at T4, the questions on interests ("I've been feeling interested in other people", "I've been interested in new things") and love ("I've been feeling loved") were not included in the eleven sub-questions that made up the final 'wellbeing_t4' variable due to their factor loadings being below the recommended minimum of 0.4 at T1, as described in 2.1.9 (Matsunaga, 2010). Cronbach's α and McDonald's ω values indicated good internal consistency (α = .86, ω = .90) for this factor.

2.1.11 Behavioral Interdependence (T2)

A CFA was run on the four items that make up the Behavioral Interdependence component of the Group Identification Scale. Henry et al. (1999) propose that these four questions load onto a single factor called Behavioral Interdependence. Indeed, this one factor model was a better fit than the null model, χ^2 (5) = 42.5, p = .001. The one factor model had a CFI of .93.

Table S2.7 Factor loadings for the one factor model of the Warwick-Edinburgh Mental Wellbeing Scale at T1 and T4

Factor	Factor loading (T1)	α, ω (T2)	Factor loading (T4)	α, ω (T4)
Wellbeing		.84, .87*		.88, .90*
"I've been feeling optimistic about the future."	.51		.56	
"I've been feeling useful."	.53		.62	
"I've been feeling relaxed."	.63		.47	
"I've been feeling interested in other people."	.38		.52	
"I've had energy to spare."	.46		.46	
"I've been dealing with problems well."	.54		.59	
"I've been thinking clearly."	.54		.58	
"I've been feeling good about myself."	.68		.72	
"I've been feeling close to other people."	.61		.70	
"I've been feeling confident."	.65		.70	
"I've been able to make up my own mind about things."	.52		.49	
"I've been feeling loved."	.39		.57	
"I've been interested in new things."	.32		.56	
"I've been feeling cheerful."	.69		.71	

* These are the Cronbach's α *and* McDonald's ω values for the final factor, which was comprised of all sub-questions with factor loadings above 0.4 at both timepoints.

However, as seen in Table S2.8, the reversed-scored question on reliance ("In this team, members don't have to rely on one another.") had a poor factor loading. This sub-question was not included in the creation of this factor ('interdependence_t2'), which was an average of participants' responses to the other three questions. Cronbach's α and McDonald's ω values indicated a potentially problematic internal consistency ($\alpha = .55$, $\omega = .58$) for this factor.

2.2 Principal Component Analyses

2.2.1 Bonding (T2)

A PCA suggested that one component be extracted from the four questions on bonding (identity fusion (Swann, Gomez, Seyle, Morales, & Huici, 2009), "How connected do you feel to the other members of your team?", "How bonded do you feel to the other members of your team?", and "How committed do you feel to the other members of your team?"); it explained 60.39% of the variance in the questions. Cronbach's α and McDonald's ω ($\alpha = .78$, $\omega = .81$) indicated good internal consistency for the bonding component ('bonding_t2').

Table S2.8 Factor loadings for the one factor model of Behavioral Interdependence at T2

**Factor**	**Factor loading**	**α, ω**
Behavioral Interdependence		.55, 58*
"In this team, members don't have to rely on one another."[1]	.15	
"All members need to contribute to achieve the team's goals."	.74	
"The team accomplishes things that no single member could achieve."	.44	
"In this team, members do not need to cooperate to complete teamtasks."[1]	.47	

[1.] Reversed scored
* This is the Cronbach's α and McDonald's ω values for the final factor, which was comprised of all sub-questions with factor loadings above 0.4.

2.2.2 Bonding (T3)

A PCA suggested that one component be extracted from the four questions on bonding (identity fusion, "How connected do you feel to the other members of your team?", "How bonded do you feel to the other members of your team?", and "How committed do you feel to the other members of your team?"); it explained 68.33% of the variance in the questions. Cronbach's α and McDonald's ω (α = .74, ω = .89) indicated good internal consistency for the bonding component ('bonding_t3').

2.2.3 Bonding (T4)

A PCA suggested that one component be extracted from the four questions on bonding (identity fusion, "How connected do you feel to the other members of your team?", "How bonded do you feel to the other members of your team?", and "How committed do you feel to the other members of your team?"); it explained 72.69% of the variance in the questions. Cronbach's α and McDonald's ω (α = .87, ω = .98) indicated good internal consistency for the bonding component ('bonding_t4').

2.2.4 Physical Discomfort (T3)

Running a PCA on the questions about experienced pain and fatigue ("How much pain did you experience overall?", and "How much fatigue did you experience overall?") yielded a component ('physical_discomfort_t3') that explained 78.61% of the variance in the two questions. Cronbach's α and McDonald's ω should not be calculated for components comprised of only two variables, but the correlation between answers to the pain and fatigue questions was strong (r = .572).

2.2.5 Performance Satisfaction (T3)

Running a PCA on the questions about satisfaction with individual and team performance relative to expectations ("How satisfied are you with your individual performance?" and "How satisfied are you with the performance of your team?") yielded a component (`peform_satisfaction_t3`) that explained 80.02% of the variance in the two questions. Cronbach's α and McDonald's ω should not be calculated for components comprised of only two variables, but the correlation between answers to the two questions was strong (r = .600).

2.2.6 Experienced Interdependence (T3)

A PCA suggested that one component be extracted from the two questions on experienced interdependence ("… how much did you need each other?" and "... how much did you help each other?"). The one component ('experienced_interdependence_t3') explained 80.28% of the variance in the question. Cronbach's α and McDonald's ω should not be calculated for components comprised of only two variables, but the correlation between answers to the two questions was strong (r = .606).

3 Measurement invariance

3.1 Warwick-Edinburgh Mental Wellbeing Scale (T1 and T4)

Regarding configural invariance, the guidelines set by Hu and Bentler (1999) for configural model fit indices suggest that the factorial structure of Wellbeing is maintained across the two time points: comparative fit index (CFI) = 1.000; Tucker-Lewis index (TLI) = 1.011; standard root mean square error of approximation ($RMSEA_{standard}$) < .001; robust root mean square error of approximation ($RMSEA_{robust}$) = .057 . The configural invariance assumption was thus met.

Regarding metric invariance, a chi-square difference test suggested that the configural invariance model was not significantly different from a model with constrained factor loadings, $\Delta\chi^2$ (10) = 4.0, p = .947. The metric invariance assumption was thus met.

Regarding scalar invariance, a chi-square difference test suggested that the metric invariance model was not significantly different from a model with constrained factor loadings and intercepts, $\Delta\chi^2$ (10) = 8.9, p = .540. The scalar invariance assumption was thus met.

Regarding residual invariance, a chi-square difference test suggested that the scalar invariance model was not significantly different from a model with constrained factor loadings, intercepts, and residuals, $\Delta\chi^2$ (11) = 8.0, p = .717. The residual invariance assumption was thus met, meaning that the Warwick-Edinburgh Mental Wellbeing Scale had strict invariance across T1 and T4.

3.2 Bonding (T2, T3, and T4)

Regarding configural invariance, the guidelines set by Hu and Bentler (1999) for configural model fit indices suggest that the same factorial structure of Bonding is maintained across the three time points: CFI = 1.000; TLI = 1.020; $RMSEA_{standard}$ < .001; $RMSEA_{robust}$ = .068. The configural invariance assumption was thus reasonably met.

Regarding metric invariance, a chi-square difference test suggested that the configural invariance model was not significantly different from a model with constrained factor loadings, $\Delta\chi^2$ (6) = 5.8, p = .450. The metric invariance assumption was thus met.

Regarding scalar invariance, a chi-square difference test suggested that the scalar invariance assumption was broken: $\Delta\chi^2$ (6) = 18.9, p = .004. In particular, the model parameters suggested that the intercept for the question "How committed do you feel to the other members of your team?" varied significantly across the three time periods. When the intercepts for this item were

estimated freely, partial scalar invariance was achieved: $\Delta\chi^2$ (4) = 3.5, p = .475 (Putnick & Bornstein, 2016).

Regarding residual invariance, a chi-square difference test suggested that the (adjusted) scalar invariance model was not significantly different from a model with constrained factor loadings, intercepts, and residuals, $\Delta\chi^2$ (8) = 13.8, p = .088. The residual invariance assumption was thus met when using the adjusted models reported above.

Both Steenkamp and Baumgartner (1998) and Vandenberg and Lance (2000) suggest that, if the majority of items in the factor are invariant, it is reasonable to carry out tests of mean differences – this is the case for the Bonding, which only had one item that was partially invariant (the item based on the question "How committed do you feel to the other members of your team?" showed partial scalar invariance).

3.3 Perceptions of Relationship – Closeness, Similarity, and Everyday Centrality (T2 & T4)

Regarding configural invariance, the guidelines set by Hu and Bentler (1999) for configural model fit indices suggest that the same factorial structure (the Closeness, Similarity, and Everyday Centrality three factor model) is maintained across the two time points: CFI = 1.000; TLI = 1.018; $RMSEA_{standard}$ < .001; $RMSEA_{robust}$ = .058. The configural invariance assumption was thus met.

Regarding metric invariance, a chi-square difference test suggested that the configural invariance model was significantly different from a model with constrained factor loadings, $\Delta\chi^2$ (11) = 24.1, p = .012. The metric invariance assumption was thus violated. In particular, the model parameters suggested that the factor loadings of three items ("How often do you talk about personal things with your team members?", "How important is your relationship with your team members?", and "How satisfied are you with your relationship to your team members?") making up the Closeness factor and two items ("My team members and I have very different values." and "My team members and I are very similar.") making up the Similarity factor were invariant across the two time points. When the factor loadings for these items were estimated freely, partial metric invariance was achieved: $\Delta\chi^2$ (6) = 1.7, p = .942 (Putnick & Bornstein, 2016).

Regarding scalar invariance, a chi-square difference test suggested that the scalar invariance assumption was broken: $\Delta\chi^2$ (16) = 44.6, p < .001. In particular, the model parameters suggested that the intercepts for two items ("How often do you talk about personal things with your team members?" and "How important is your relationship with your team members?") making up the Closeness factor, one item making up the Similarity factor ("My team members and I have very different values."), and one item ("How often do you talk to your team members?") making up the Everyday Centrality factor were invariant across the two time points. When the intercepts for these items were estimated freely, partial scalar invariance was achieved: $\Delta\chi^2$ (7) = 5.1, p = .649 (Putnick & Bornstein, 2016).

Regarding residual invariance, a chi-square difference test suggested that the scalar invariance assumption was broken: $\Delta\chi^2$ (14) = 25.4, p = .031. In particular, the model parameters suggested noninvariance across the two time points for the residuals of one item ("How often do you talk about personal things with your team members?") making up the Closeness factor, one item ("My team members and I are very similar.") making up the Similarity factor, and one time ("How

central are your team members to your everyday life?") making up the Everyday Centrality factor. When the intercepts for these items were estimated freely, partial residual invariance was achieved: $\Delta\chi^2$ (11) = 13.1, p = .288 (Putnick & Bornstein, 2016).

Analyses revealed partial measurement invariance for the Closeness, Similarity, and Everyday Centrality factors. Although there are no universally accepted benchmarks for continuing with tests of mean differences (as done in our analyses) for factors displaying partial invariance (Putnick & Bornstein, 2016), some guidelines have been suggested. Again, both Steenkamp and Baumgartner (1998) and Vandenberg and Lance (2000) suggest that, if the majority of items in the factor are invariant, it is reasonable to carry out tests of mean differences. Applying these guidelines to the factors making up the Perceptions of Relationship scale, only the Closeness factor would potentially have a problematic amount of measurement invariance, as three of the six items that made up the factor showed metric invariance. This suggests that the relationship of these items to the Closeness factor may have changed from T2 to T4. However, Steinmetz (2013) have used Monte-Carlo simulation to show that metric noninvariance had a negligible on the results of mean difference tests for latent factors. Thus, we maintain report the result of the change in Closeness from T2 to T4 in the main manuscript, but flag that these results should be interpreted with caution, given indications of measurement invariance.

3.4 Mood – Tension, Vigor, Confusion, Fatigue, Depression, and Anger (T2 & T3)

Regarding configural invariance, the guidelines set by Hu and Bentler (1999) for configural model fit indices suggest that the same factorial structure (the Tension, Vigor, Confusion, Fatigue, Depression, and Anger six factor model) is maintained across the two time points: CFI = 1.000; TLI = 1.004; $RMSEA_{standard}$ < .001; $RMSEA_{robust}$ = .058. The configural invariance assumption was thus met.

Regarding metric invariance, a chi-square difference test suggested that the configural invariance model was significantly different from a model with constrained factor loadings, $\Delta\chi^2$ (17) = 33.8, p = .009. The metric invariance assumption was thus violated. In particular, the model parameters suggested noninvariance across the two time points for the factor loadings of one item ("Anxious") making up the Tension factor, one item ("Alert") making up the Vigor factor, two items ("Muddled" and "Uncertain") making up the Confusion factor, two items ("Exhausted" and "Sleepy") making up the Fatigue factor, and one item ("Unhappy") making up the Depression factor. When the factor loadings for these items were estimated freely, partial metric invariance was achieved: $\Delta\chi^2$ (10) = 9.5, p = .486 (Putnick & Bornstein, 2016).

Regarding scalar invariance, a chi-square difference test suggested that the scalar invariance assumption was broken: $\Delta\chi^2$ (24) = 110.7, p < .001. In particular, the model parameters suggested noninvariance across the two time points for the intercepts of two items ("Panicky" and "Nervous") making up the Tension factor, one item ("Lively") making up the Vigor factor, two items ("Mixed-up" and "Uncertain") making up the Confusion factor, and all four items ("Worn out", "Sleepy", "Exhausted", and "Tired") making up the Fatigue factor. When the intercepts for these items were estimated freely, partial scalar invariance was achieved: $\Delta\chi^2$ (8) = 14.0, p = .083 (Putnick & Bornstein, 2016).

Regarding residual invariance, a chi-square difference test suggested that the scalar invariance assumption was broken: $\Delta\chi^2$ (23) = 72.8, p < .001. In particular, the model parameters suggested noninvariance across the two time points for the residuals of one item ("Anxious") making up the Tension factor, two items ("Lively" and "Alert") making up the Vigor factor, two items ("Confused" and "Mixed-up") making up the Confusion factor, and three items ("Worn out", "Sleepy", and "Exhausted") making up the Fatigue factor. When the intercepts for these items were estimated freely, partial residual invariance was achieved: $\Delta\chi^2$ (15) = 24.3, p = .061 (Putnick & Bornstein, 2016).

Analyses revealed only partial measurement invariance for several of the mood factors. In particular, both the Confusion and Fatigue factors had only partial metric, scalar, and residual invariance, while the Tension and Vigor factors had only partial scalar and residual invariance. Previous research has shown that it is reasonable to carry out tests of mean differences of a latent factors if the majority of items in the factor are invariant (Steenkamp & Baumgartner, 1998; Vandenberg & Lance, 2000). This is not the case for the Confusion, Fatigue, Tension, or Vigor factors, all of which have invariance of some type in half or more of their items. Mean difference tests for the Confusion, Fatigue, Tension, and Vigor factors are thus not reported in the main text.

4 Main analyses – model statistics

This section presents tables with full model results for all analyses reported in the main manuscript.

Table S4.1 Change in Anger over time (T2 to T3)

**Variable**	***b***	***SE***	***t* (*df*)**	***p***
Intercept	0.086	0.052	1.64 (147.4)	.102
Time (T2 to T3)	0.15	0.043	3.52 (162.9)	.001
Sex (male)	0.061	0.056	1.10 (171.8)	.274
**Random part**	**Variance**	***SD***		
Participant	0.023	0.150		
Team	0.032	0.180		
R^2_m = 0.031, R^2_c = 0.285				

Table S4.2 Change in Depression over time (T2 to T3)

**Variable**	**b**	***SE***	***t* (*df*)**	***p***
Intercept	0.098	0.052	1.89 (173.8)	.061
Time (T2 to T3)	0.131	0.048	2.73 (301.5)	.007
Sex (male)	0.053	0.055	0.98 (227.0)	.328
**Random part**	**Variance**	***SD***		
Participant	0	0		
Team	0.024	0.155		
$R^2_m = 0.022$, $R^2_c = 0.128$				

Table S4.3 Change in Fatigue over time (T2 to T3)

**Variable**	***b***	***SE***	***t* (*df*)**	***p***
Intercept	0.806	0.09	8.97 (168.5)	< .001
Time (T2 to T3)*	2.08	0.075	27.55 (173.7)	< .001
Sex (male)	-0.153	0.101	-1.52 (168.2)	.131
**Random part**	**Variance**	**SD**		
Participant	0.168	0.409		
Team	0.030	0.172		
$R^2_m = 0.612$, $R^2_c = 0.725$				

* The Fatigue factor had a significant amount of measurement noninvariance from T2 to T3, making this mean difference test potentially problematic (see SOM 3.4).

Table S4.4 Change in Tension over time (T2 to T3)

Variable	*b*	*SE*	*t* (*df*)	*p*
Intercept	1.292	0.063	20.53 (302.0)	< .001
Time (T2 to T3)*	-0.871	0.065	-13.45 (188.7)	< .001
Sex (male)	-0.31	0.068	-4.57 (188.2)	< .001
**Random part**	**Variance**	***SD***		
Participant	0.015	0.122		
Team	0	0		

$R^2_m = 0.359$, $R^2_c = 0.384$

* The Tension factor had a significant amount of measurement noninvariance from T2 to T3, making this mean difference test potentially problematic (see SOM 3.4).

Table S4.5 Change in Vigor over time (T2 to T3)

Variable	*b*	*SE*	*t* (*df*)	*p*
Intercept	2.314	0.098	23.50 (164.6)	< .001
Time (T2 to T3)*	-0.448	0.072	-6.20 (176.6)	< .001
Sex (male)	0.159	0.110	1.44 (192.4)	.151
**Random part**	**Variance**	***SD***		
Participant	0.209	0.457		
Team	0.095	0.308		

$R^2_m = 0.069$, $R^2_c = 0.452$

* The Vigor factor had a significant amount of measurement noninvariance from T2 to T3, making this mean difference test potentially problematic (see SOM 3.4).

Table S4.6 Change in Confusion over time (T2 to T3)

**Variable**	***b***	***SE***	***t* (*df*)**	***p***
(Intercept)	0.453	0.055	8.21 (133.6)	< .001
Time (T2 to T3)*	-0.018	0.041	-0.44 (161.6)	.663
Sex (male)	-0.139	0.062	-2.25 (170.3)	.026
**Random part**	**Variance**	**SD**		
Participant	0.069	0.263		
Team	0.027	0.165		

$R^2_m = 0.020$, $R^2_c = 0.424$

* The Confusion factor had a significant amount of measurement noninvariance from T2 to T3, making this mean difference test potentially problematic (see SOM 3.4).

Table S4.7 Change in Closeness over time (T2 to T4)

**Variable**	***b***	***SE***	***t* (*df*)**	***p***
Intercept	5.644	0.115	49.27 (156.7)	< .001
Time (T2 to T4)	-0.330	0.068	-4.86 (140.1)	< .001
Sex (male)	0.022	0.130	0.17 (185.8)	.867
**Random part**	**Variance**	**SD**		
Participant	0.324	0.569		
Team	0.248	0.498		

$R^2_m = 0.03$, $R^2_c = 0.649$

Table S4.8 Change in Everyday Centrality over time (T2 to T4)

**Variable**	***b***	***SE***	***t* (*df*)**	***p***
Intercept	4.856	0.167	29.01 (162.4)	< .001
Time (T2 to T4)	-0.367	0.077	-4.74 (136.7)	< .001
Sex (male)	0.173	0.187	0.93 (189.0)	.355
**Random part**	**Variance**	***SD***		
Participant	0.726	0.852		
Team	0.728	0.854		
$R^2_m = 0.022$, $R^2_c = 0.786$				

Table S4.9 Change in Similarity over time (T2 to T4)

**Variable**	***b***	***SE***	***t* (*df*)**	***p***
Intercept	4.910	0.139	35.30 (161.2)	< .001
Time (T2 to T4)	-0.040	0.076	-0.520 (140.4)	.601
Sex (male)	0.022	0.162	0.130 (187.0)	.893
**Random part**	**Variance**	***SD***		
Participants	0.622	0.788		
Team	0.309	0.556		
$R^2_m < 0.001$, $R^2_c = 0.699$				

Table S4.10 Change in Well-being over time (T1 to T4)

Variable	*b*	*SE*	*t* (*df*)	*p*
Intercept	3.638	0.054	66.77 (161.6)	< .001
Time (T1 to T4)	0.079	0.034	2.33 (166.2)	.021
Sex (male)	0.223	0.067	3.35 (181.6)	.001
**Random part**	**Variance**	***SD***		
Participant	0.138	0.371		
Team	0.017	0.129		
R^2_m = 0.052, R^2_c = 0.650				

Table S4.11 Effects of Performance Satisfaction (T3) on Well-being increase (T1 to T4)*

Variable	*b*	*SE*	*t* (*df*)	*p*
Intercept	0.019	0.056	0.21 (136.0)	.837
Performance Satisfaction (T3)	0.113	0.041	2.79 (136.0)	.006
Sex (male)	0.056	0.074	0.80 (136.0)	.423
**Random part**	**Variance**	***SD***		
Team	0.004	0.067		
R^2_m = 0.050, R^2_c = 0.301				

* To avoid a singular fit during model estimation, this model was fit using maximum a posteriori estimation. Estimate degrees of freedom are taken from the model with a singular fit (which used maximum likelihood estimation), as maximum a posteriori estimation in R does not provide degree of freedom calculations.

Table S4.12 Effects of Bonding (T3) on Well-being increase (T1 to T4)

**Variable**	***b***	***SE***	***t* (*df*)**	***p***
Intercept	0.026	0.063	0.41 (136.0)	.681
Bonding (T3)	0.107	0.038	2.81 (136.0)	.006
Sex (male)	0.048	0.079	0.60 (136.0)	.656
**Random part**	**Variance**	***SD***		
Team	0.051	0.226		

$R^2_m = 0.051$, $R^2_c = 0.301$

* To avoid a singular fit during model estimation, this model was fit using maximum a posteriori estimation. Estimate degrees of freedom are taken from the model with a singular fit (which used maximum likelihood estimation), as maximum a posteriori estimation in R does not provide degree of freedom calculations.

Table S4.13 Change in Bonding over time (T2 to T3)

**Variable**	***b***	***SE***	***t* (*df*)**	***p***
Intercept	5.331	0.097	54.88 (146.0)	< .001
Time (T2 to T3)	0.394	0.056	7.00 (159.4)	< .001
Sex (male)	-0.116	0.112	-1.04 (186.6)	.302
**Random part**	**Variance**	***SD***		
Participant	0.325	0.570		
Team	0.103	0.321		

$R^2_m = 0.057$, $R^2_c = 0.647$

Table S4.14 Change in Bonding over time (T2 to T4)

**Variable**	***b***	***SE***	***t* (*df*)**	***p***
Intercept	5.117	0.117	43.69 (153.2)	< .001
Time (T2 to T4)	-0.212	0.064	-3.31 (134.5)	.001
Sex (male)	0.164	0.136	1.20 (180.9)	.231
**Random part**	**Variance**	***SD***		
Participant	0.434	0.659		
Team	0.224	0.473		
R^2_m = 0.019, R^2_c = 0.704				

Table S4.15 Effects of change in Bonding (T2 to T3) on Well-being increase (T1 to T4)*

**Variable**	***b***	***SE***	***t* (*df*)**	***p***
Intercept	0.059	0.068	0.87 (119.0)	.386
Bonding change (T2 to T3)	0.090	0.049	1.83 (119.0)	.070
Sex (male)	0.038	0.087	0.44 (119.0)	.664
**Random part**	**Variance**	***SD***		
Team	0.002	0.049		
R^2_m = 0.024, R^2_c = 0.036				

* To avoid a singular fit during model estimation, this model was fit using maximum a posteriori estimation. Estimate degrees of freedom are taken from the model with a singular fit (which used maximum likelihood estimation), as maximum a posteriori estimation in R does not provide degree of freedom calculations.

Table S4.16 Effects of Bonding (T2) on Well-being increase (T1 to T4)*

**Variable**	***b***	***SE***	***t***	***p***
Intercept	0.826	0.069	1.20 (122.0)	.232
Bonding (T2)	0.009	0.043	0.23 (122.0)	.820
Sex (male)	0.034	0.084	0.430 (122.0)	.668
**Random part**	**Variance**	***SD***		
Team	0.057	0.238		
R^2_m = 0.001, R^2_c = 0.268				

* To avoid a singular fit during model estimation, this model was fit using maximum a posteriori estimation. Estimate degrees of freedom are taken from the model with a singular fit (which used maximum likelihood estimation), as maximum a posteriori estimation in R does not provide degree of freedom calculations.

Table S4.17 Effects of Physical Discomfort on Bonding (T3)

**Variable**	***b***	***SE***	***t* (*df*)**	***p***
Intercept	0.111	0.116	0.96 (111.8)	.339
Physical Discomfort	0.240	0.071	3.41 (183.4)	.001
Sex (male)	-0.181	0.145	-1.25 (175.4)	.212
**Random part**	**Variance**	***SD***		
Team	0.079	0.281		
R^2_m = 0.072, R^2_c = 0.152				

Table S4.18 Effects of Experienced Interdependence on Bonding (T3)[*]

**Variable**	***b***	***SE***	***t* (*df*)**	***p***
Intercept	0.027	0.108	0.25 (190.0)	.802
Experienced Interdependence	0.528	0.062	8.52 (190.0)	< .001
Sex (male)	-0.056	0.129	-0.44 (190.0)	.662
**Random part**	**Variance**	***SD***		
Team	0.165	0.406		

$R^2_m = 0.279$, $R^2_c = 0.440$

[*] To avoid a singular fit during model estimation, this model was fit using maximum a posteriori estimation. Estimate degrees of freedom are taken from the model with a singular fit (which used maximum likelihood estimation), as maximum a posteriori estimation in R does not provide degree of freedom calculations.

Table S4.19 Effects of Team Performance Relative to Expectations on Bonding (T3)

**Variable**	***b***	***SE***	***t* (*df*)**	***p***
Intercept	-1.188	0.308	-3.85 (175.8)	< .001
Team Performance Relative to Expectations	0.097	0.048	2.02 (188.0)	.045
Sex (male)	-0.154	0.142	-1.09 (174.6)	.279
Individual Performance Relative to Expectations	0.093	0.043	2.18 (190.7)	.031
**Random part**	**Variance**	***SD***		
Team	0.068	0.261		

$R^2_m = 0.111$, $R^2_c = 0.178$

Table S4.20 Effects of Received Emotional Support on Bonding (T3)

**Variable**	***b***	***SE***	***t* (*df*)**	***p***
Intercept	-2.952	0.308	-9.59 (189.5)	< .001
Received Emotional Support	0.745	0.071	10.50 (190.3)	< .001
Sex (male)	0.006	0.119	0.050 (173.7)	.959
**Random part**	**Variance**	**SD**		
Team	0.020	0.141		
$R^2_m = 0.370$, $R^2_c = 0.390$				

Table S4.21 Effects of Received Esteem Support on Bonding (T3)*

**Variable**	***b***	***SE***	***t* (*df*)**	***p***
Intercept	-2.799	0.323	-8.65 (193.0)	< .001
Received Esteem Support	0.688	0.073	9.47 (193.0)	< .001
Sex (male)	-0.006	0.126	-0.05 (193.0)	.962
**Random part**	**Variance**	***SD***		
Team	0.165	0.406		
$R^2_m = 0.317$, $R^2_c = 0.474$				

* To avoid a singular fit during model estimation, this model was fit using maximum a posteriori estimation. Estimate degrees of freedom are taken from the model with a singular fit (which used maximum likelihood estimation), as maximum a posteriori estimation in R does not provide degree of freedom calculations.

Table S4.22 Effects of Behavioral Interdependence on Bonding (T2)

**Variable**	***b***	***SE***	***t* (*df*)**	***p***
Intercept	-1.593	0.453	-3.52 (154.0)	0.001
Behavioral Interdependence	0.273	0.071	3.83 (149.0)	< .001
Sex (male)	-0.086	0.159	-0.54 (145.7)	0.590
**Random part**	**Variance**	***SD***		
Team	0.171	0.413		
$R^2_m = 0.084$, $R^2_c = 0.257$				

Table S4.23 Effects of Perceived Emotional Support on Bonding (T2)

**Variable**	***b***	***SE***	***t* (*df*)**	***p***
Intercept	-3.196	0.441	-7.24 (157.1)	< .001
Perceived Emotional Support	0.620	0.081	7.70 (157.6)	< .001
Sex (male)	0.037	0.142	0.26 (140.4)	.797
**Random part**	**Variance**	***SD***		
Team	0.079	0.282		
$R^2_m = 0.277$, $R^2_c = 0.358$				

Table S4.24 Effects of Perceived Esteem Support on Bonding (T2)

**Variable**	***b***	***SE***	***t* (*df*)**	***p***
Intercept	-3.255	0.458	-7.10 (149.4)	< .001
Perceived Esteem Support	0.633	0.084	7.53 (150.1)	< .001
Sex (male)	0.003	0.142	0.02 (136.8)	.982
**Random part**	**Variance**	***SD***		
Team	0.086	0.293		
$R^2_m = 0.274$, $R^2_c = 0.361$				

Table S4.25 Effects of Bonding (T2) on Physical Discomfort

**Variable**	***b***	***SE***	***t* (*df*)**	***p***
Intercept	-2.935	0.716	-4.10 (148.8)	< .001
Bonding (T2)	0.039	0.077	0.51 (149.8)	.610
Sex (male)	-0.155	0.155	-1.00 (135.7)	.319
Effort	0.342	0.075	4.56 (147.1)	< .001
**Random part**	**Variance**	***SD***		
Team	0.131	0.362		
$R^2_m = 0.148$, $R^2_c = 0.286$				

Table S4.26 Effects of Perceived Emotional Support (T2) on Physical Discomfort

**Variable**	***b***	***SE***	***t* (*df*)**	***p***
Intercept	-3.348	0.726	-4.62 (150.0)	< .001
Perceived Emotional Support (T2)	0.123	0.092	1.34 (149.4)	.182
Sex (male)	-0.132	0.155	-0.85 (136.8)	.398
Effort	0.317	0.076	4.17 (146.1)	< .001
**Random part**	**Variance**	***SD***		
Team	0.132	0.363		
$R^2_m = 0.157$, $R^2_c = 0.296$				

Table S4.27 Effects of Perceived Esteem Support (T2) on Physical Discomfort

**Variable**	***b***	***SE***	***t* (*df*)**	***p***
Intercept	-3.159	0.731	-4.32 (150.0)	< .001
Perceived Esteem Support (T2)	0.052	0.096	0.54 (147.3)	.594
Sex (male)	-0.150	0.156	-0.96 (135.7)	.336
Effort	0.337	0.077	4.37 (147.7)	< .001
**Random part**	**Variance**	***SD***		
Team	0.134	0.366		
$R^2_m = 0.148$, $R^2_c = 0.289$				

Table S4.28 Effects of Experienced Interdependence on changes in Bonding (T2 to T3)

**Variable**	***b***	***SE***	***t* (*df*)**	***p***
Intercept	0.209	0.116	1.60 (95.3)	.074
Experienced Interdependence	0.177	0.072	2.44 (150.3)	.016
Sex (male)	-0.094	0.147	-0.64 (133.2)	.523
**Random part**	**Variance**	***SD***		
Team	0.082	0.287		
$R^2_m = 0.027$, $R^2_c = 0.141$				

Table S4.29 Effects of Physical Discomfort on changes in Bonding (T2 to T3)

**Variable**	***b***	***SE***	***t* (*df*)**	***p***
Intercept	0.264	0.119	2.22 (91.2)	.029
Physical Discomfort	0.028	0.073	0.39 (149.0)	.697
Sex (male)	-0.172	0.148	-1.16 (131.7)	.248
**Random part**	**Variance**	***SD***		
Team	0.103	0.321		

$R^2_m = 0.011$, $R^2_c = 0.147$

Table S4.30 Effects of Bonding (T3) on Performance Satisfaction*

**Variable**	***b***	***SE***	***t* (*df*)**	***p***
Intercept	0.085	0.115	0.74 (193.0)	.459
Bonding (T3)	0.408	0.661	6.17 (193.0)	< .001
Sex (male)	-0.105	0.139	-0.76 (193.0)	.449
**Random part**	**Variance**	***SD***		
Team	0.184	0.429		

$R^2_m = 0.166$, $R^2_c = 0.342$

* To avoid a singular fit during model estimation, this model was fit using maximum a posteriori estimation. Estimate degrees of freedom are taken from the model with a singular fit (which used maximum likelihood estimation), as maximum a posteriori estimation in R does not provide degree of freedom calculations.

Table S4.31 Experienced Interdependence by Physical Discomfort interaction on Bonding (T3)[*]

**Variable**	***b***	***SE***	***t***	***p***
Intercept	-0.002	0.111	-0.02 (188.0)	.987
Experienced Interdependence	0.512	0.069	7.37 (188.0)	< .001
Physical Discomfort	0.115	0.065	1.77 (188.0)	.077
Sex (male)	-0.025	0.132	-0.19 (188.0)	.582
Experienced Interdependence × Physical Discomfort	0.019	0.050	0.38 (188.0)	.703
**Random part**	**Variance**	***SD***		
Team	0.163	0.404		

$R^2_m = 0.289$, $R^2_c = 0.448$

[*] To avoid a singular fit during model estimation, this model was fit using maximum a posteriori estimation. Estimate degrees of freedom are taken from the model with a singular fit (which used maximum likelihood estimation), as maximum a posteriori estimation in R does not provide degree of freedom calculations.

Table S4.32 Received Emotional Support by Physical Discomfort interaction on Bonding (T3)*

**Variable**	***b***	***SE***	***t***	***p***
Intercept	-2.675	0.311	-8.60 (186.1)	< .001
Received Emotional Support	0.687	0.071	9.61 (185.8)	< .001
Physical Discomfort	0.545	0.262	2.08 (187.9)	.039
Sex (male)	-0.017	0.118	-0.14 (167.7)	.885
Received Emotional Support × Physical Discomfort	-0.110	0.067	-1.64 (188.0)	.103
**Random part**	**Variance**	***SD***		
Team	0.005	0.068		

$R^2_m = 0.396$, $R^2_c = 0.401$

Table S4.33 Received Esteem Support by Physical Discomfort interaction on Bonding (T3)[*]

**Variable**	***b***	***SE***	***t***	***p***
Intercept	-2.477	0.340	-7.29 (188.0)	< .001
Received Esteem Support	0.619	0.076	8.18 (188.0)	< .001
Physical Discomfort	0.451	0.252	1.79 (188.0)	.074
Sex (male)	-0.036	0.127	-0.28 (188.0)	.779
Received Esteem Support × Physical Discomfort	-0.081	0.062	-1.30 (188.0)	.194
**Random part**	**Variance**	**SD**		
Team	0.150	0.387		

$R^2_m = 0.337$, $R^2_c = 0.483$

[*] To avoid a singular fit during model estimation, this model was fit using maximum a posteriori estimation. Estimate degrees of freedom are taken from the model with a singular fit (which used maximum likelihood estimation), as maximum a posteriori estimation in R does not provide degree of freedom calculations.

Table S4.34 Effects of Perceived Emotional Support on Collective Efficacy; pathway *a* of mediation analyses

**Variable**	***b***	***SE***	***t* (*df*)**	***p***
Intercept	3.692	0.741	4.98 (157.4)	< .001
Perceived Emotional Support	0.655	0.135	4.85 (157.8)	< .001
Sex (male)	0.737	0.239	3.08 (141.0)	.002
**Random part**	**Variance**	**SD**		
Team	0.266	0.515		

$R^2_m = 0.150$, $R^2_c = 0.263$

Table S4.35 Effects of Collective Efficacy on perceptions of Threat, while controlling for Perceived Emotional Support; pathway *b* of mediation analyses

**Variable**	***b***	***SE***	***t* (*df*)**	***p***
Intercept	1.016	0.54	1.88 (145.3)	.062
Collective Efficacy	-0.023	0.054	-0.42 (149.1)	.674
Perceived Emotional Support	-0.098	0.099	-0.99 (151.7)	.324
Sex (male)	-0.592	0.163	-3.64 (104.5)	< .001
**Random part**	**Variance**	**SD**		
Team	0.006	0.074		
$R^2_m = 0.087$, $R^2_c = 0.093$				

Table S4.36 Effects of Perceived Esteem Support on Collective Efficacy; pathway *a* of mediation analyses

**Variable**	***b***	***SE***	***t* (*df*)**	***p***
Intercept	3.720	0.774	4.80 (152.2)	< .001
Perceived Esteem Support	0.653	0.142	4.59 (153.1)	< .001
Sex (male)	0.695	0.241	2.88 (142.3)	.005
**Random part**	**Variance**	**SD**		
Team	0.349	0.591		
$R^2_m = 0.142$, $R^2_c = 0.288$				

Table S4.37 Effects of Collective Efficacy on perceptions of Threat, while controlling for Perceived Esteem Support; pathway *b* of mediation analyses

**Variable**	***b***	***SE***	***t* (*df*)**	***p***
Intercept	1.594	0.543	2.94 (136.4)	.004
Collective Efficacy	0.002	0.053	0.03 (147.6)	.976
Perceived Esteem Support	-0.240	0.099	-2.44 (130.3)	.016
Sex (male)	-0.640	0.159	-4.04 (107.4)	< .001
**Random part**	**Variance**	**SD**		
Team	0.004	0.063		
$R^2_m = 0.115$, $R^2_c = 0.119$				

5 Main analyses – model figures

This section presents plots of model estimates of the statistically significant predictors of Performance Satisfaction and Bonding (T3). It also presents plots of (statistically non-significant) interactions between the effects of Physical Discomfort and Received Emotional Support on Bonding (T3) and the effects of Physical Discomfort and Received Esteem Support on Bonding (T3). All models control for the effect of participant Sex.

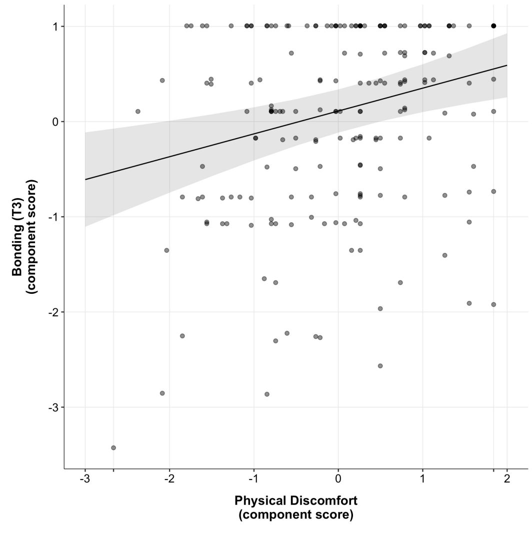

Figure S5.1 Plot of estimated effect (with shaded 95% CI) of Physical Discomfort on Bonding (T3).

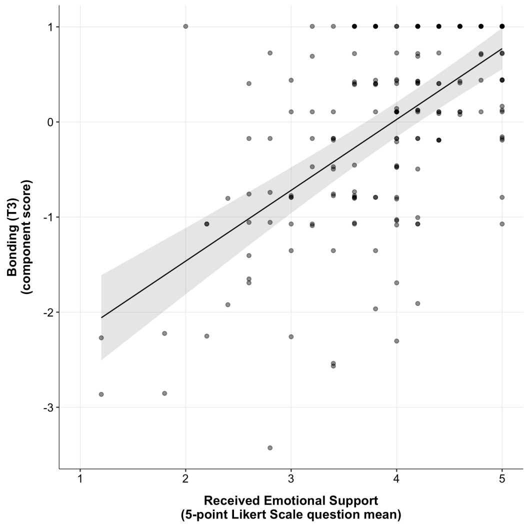

Figure S5.2 Plot of estimated effect (with shaded 95% CI) of Received Emotional Support on Bonding (T3).

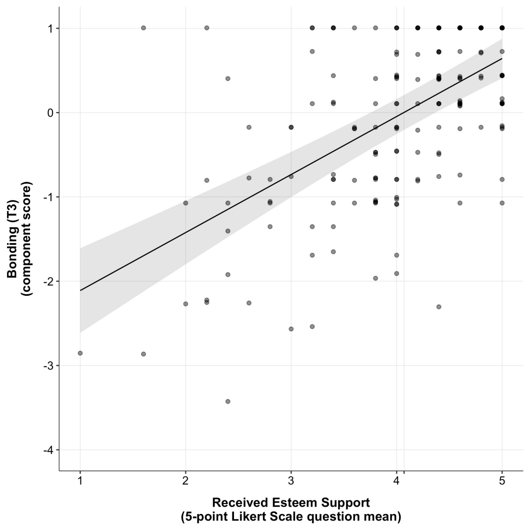

Figure S5.3 Plot of estimated effect (with shaded 95% CI) of Received Esteem Support on Bonding (T3).

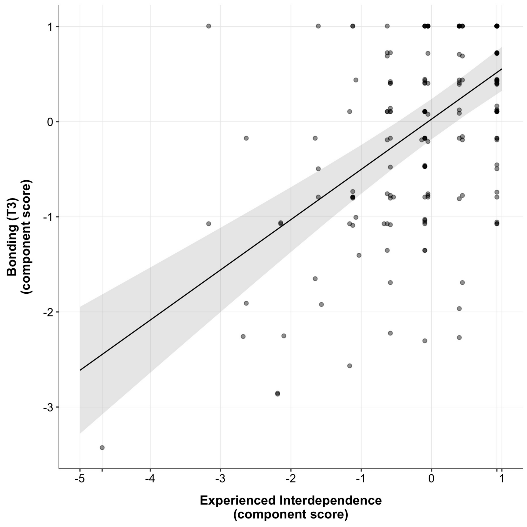

Figure S5.4 Plot of estimated effect (with shaded 95% CI) of Experienced Interdependence on Bonding (T3).

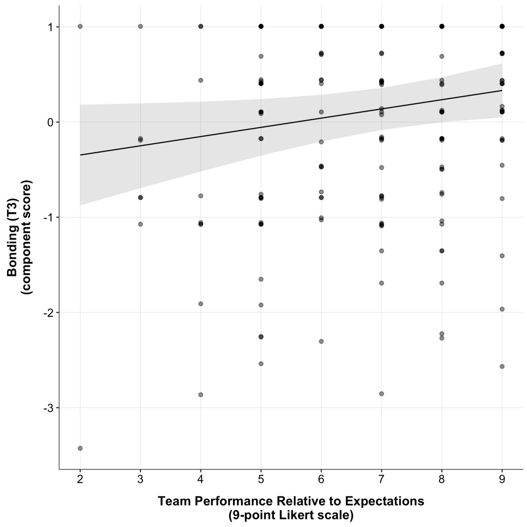

Figure S5.5 Plot of estimated effect (with shaded 95% CI) of subjective ratings of Team Performance Relative to Expectations on Bonding (T3), while controlling for subjective ratings of Individual Performance Relative to Expectations.

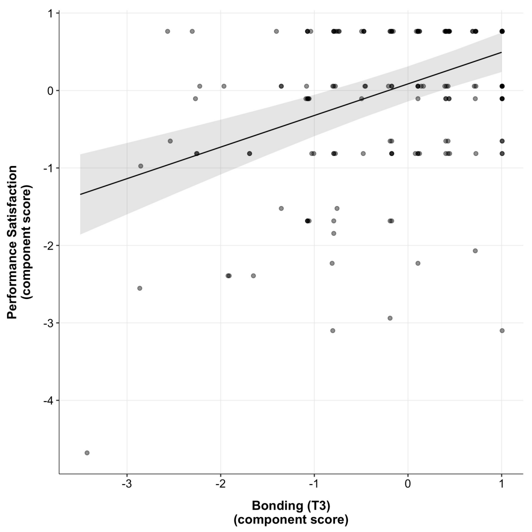

Figure S5.6 Plot of estimated effect (with shaded 95% CI) of Bonding (T3) on Performance Satisfaction.

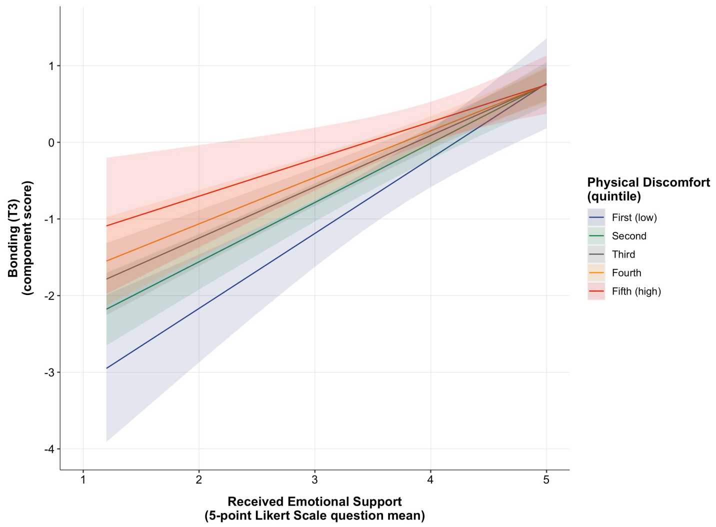

Figure S5.7 Plot of estimated effect (with shaded 95% CI) of Received Emotional Support on Bonding (T3) at each quintile of Physical Discomfort (the fifth quintile contains the participants who reported the highest levels of Physical Discomfort).

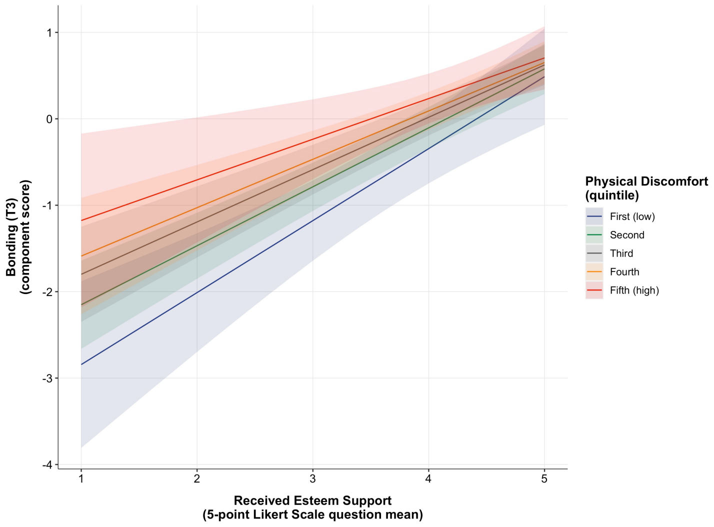

Figure S5.8 Plot of estimated effect (with shaded 95% CI) of Received Esteem Support on Bonding (T3) at each quintile of Physical Discomfort (the fifth quintile contains the participants who reported the highest levels of Physical Discomfort).

6 Model assumption checks

Model assumptions were checked according to the methods published by Snijders & Bosker (2012). The assumption checks for all models can be acquired by running the main analysis script, which will produce a folder for each model that will contain test statistics and plots for all assumption checks. For all models in the study that had significant effects with regard to our hypotheses, the assumptions were either met or broken in way that did not bias results.

Given the similarity between models used in the study, and uniformity of the justifications used for ignoring broken assumptions, we formally report only the assumption checks for the multilevel model showing decreases in Everyday Centrality from T2 to T4, and the level-one linearity check for the model testing the effects of subjective ratings of Team Performance Relative to Expectations on Bonding at T3, controlling for subjective ratings of Individual Performance Relative to Expectations, since this check is for models with continuous level-one covariates.

We chose the assumption checks for this model because its methods and interpretations are representative of those used for all other model assumptions check that were carried out for this study. Again, all information needed for the assumption checks not reported here are available through running the main analysis script.

6.1 Level-one residual homoscedasticity

Level-one homoscedasticity was assessed by observing between-group (i.e., team) differences in level-one residual variance, acquired through running separate OLS regressions on the data from

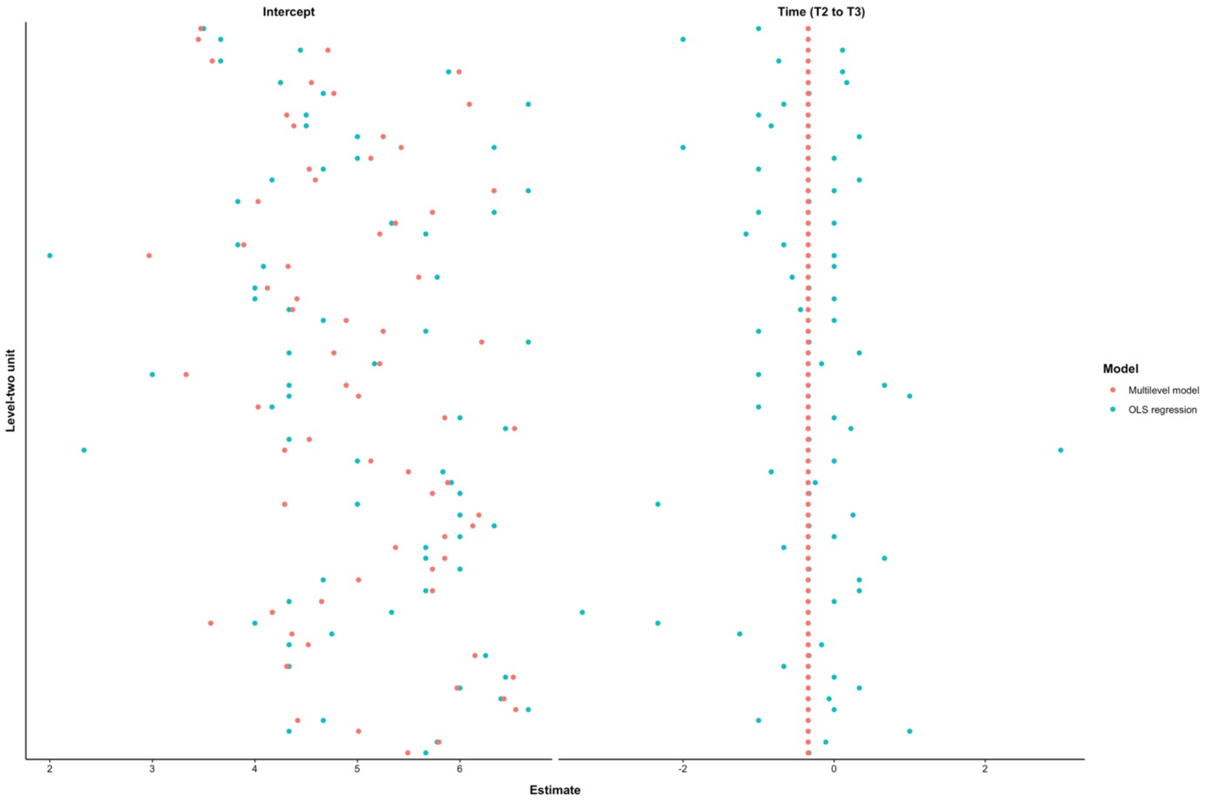

Figure S5.1 OLS coefficient estimates for each team (blue) versus the posterior means for each team in the multilevel model (pink). Both models used the time (T1 to T4) as the predictor variable, and Wellbeing change as the outcome.

participants included in the model. The variability of posterior means estimated by the multilevel model should be similar to variability of coefficients estimated by the separate OLS regressions.

Well-specified models should also have constant (i.e., homoscedastic) level-one residual variance with residual dispersion measures that are normally distributed. This can be tested using the method of Raudenbush & Bryk (2002), which tests a (squared) standardized residual dispersion measure, d, against a null hypothesis of homoscedastic level-one residual variance; *d* will have a Gaussian distribution when there is level-one homoscedasticity. When testing constant level-one residual variance with *d*, it has been suggested to only include data from relatively large level-two groups to avoid bias (Snijders & Bosker, 2012). In this case, only those teams with participant response counts at least one standard deviation above the mean were included.

Figure S5.1 shows that the variability of posterior means (for random intercepts – random slopes were not included due to model convergence failure) estimated by the multilevel model are similar to the variability of coefficients estimated by the separate OLS regressions, indicating level-one homoscedasticity (Snijders & Bosker, 2012).

Nevertheless, the null hypothesis of level-one homoscedasticity was rejected for this data, $H = 29.23$, $df = 14$, $p < .001$, indicating level-one heteroscedasticity (as the standardized residual dispersion measure, *d*, was not Gaussian). However, the Q-Q plot of *d* reveals that it is close to

(a) (b)

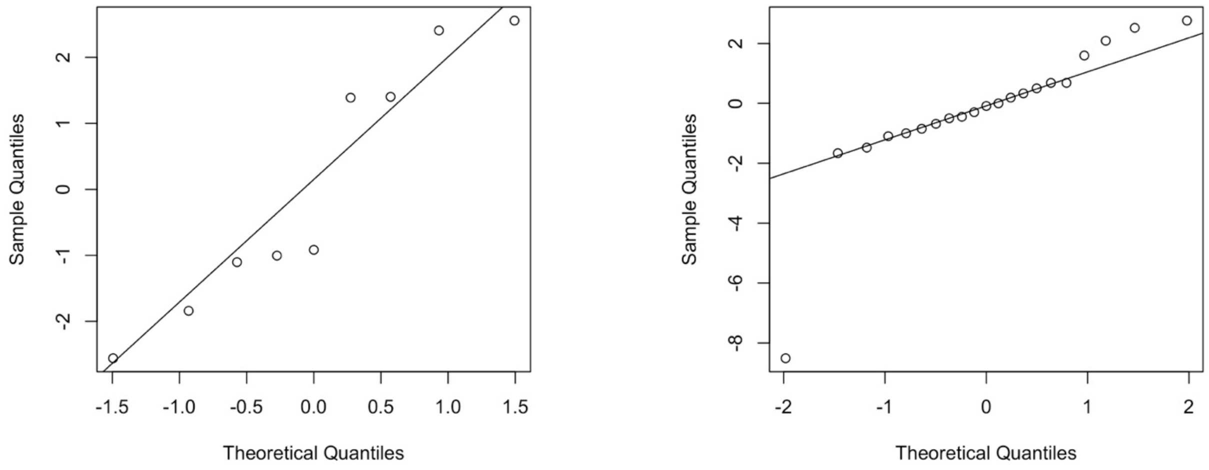

Figure S5.2 (a) Q-Q plot of the standardized residual dispersion measure, d and (b) level-one residuals for multilevel model on perceived energy levels.

being normally distributed (see Figure S5.2a). The value of *H* also depends on the normality of the level-one residuals. Heavier-tailed distributions of level-one residuals will cause inflated values of *H*, even if the residuals have (relatively) constant variance. The level-one residuals for the current model are indeed heavily tailed (see Figure S5.2b).

Given that *d* is relatively normally distributed, it can be assumed that the model has an unproblematic amount of heteroscedasticity (Snijders & Bosker, 2012).

6.2 Level-one residual normality

Normal probability plots of standardized OLS residuals were used to check level-one residual normality (Snijders & Bosker, 2012). Figure S5.2b shows that the normality assumption has been violated, due to longer tails than what would be expected with the normal distribution; specifically, the residuals display positive skew. However, research has demonstrated that fixed effects estimates are robust to violations of the normality assumption; multilevel models with non-normally distributed level-one residuals do not produced biased fixed effect parameter estimates (Seco, García, García, & Rojas, 2013).

6.3 Level-one linearity

Plots of unstandardized ordinary least squares (OLS) residuals against level-one explanatory variables (covariates) were used to check the linearity of fixed effects (Snijders & Bosker, 2012). For the model testing the effects of subjective ratings of Team Performance Relative to Expectations on Bonding at T3, controlling for subjective ratings of Individual Performance Relative to Expectations, level-one linearity was confirmed by plots of the unstandardized OLS residuals against the only relevant level-one predictor – subjective ratings of Individual

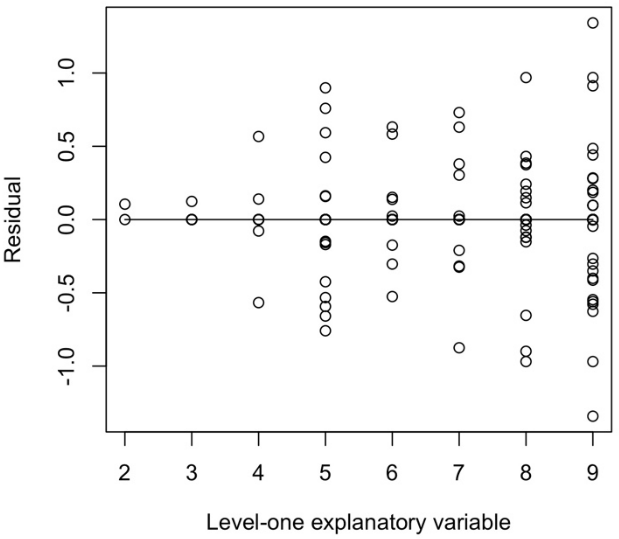

Figure S5.3 Unstandardized OLS residuals with LOWESS (locally weighted scatterplot smoothing) line for the only level-one covariate: subjective ratings of Individual Performance Relative to Expectations.

Performance Relative to Expectations. Residuals were centered around 0 at all levels of this predictor (see Figure S5.3).

6.4 Level-two residual homoscedasticity

To check level-two residual homoscedasticity, it is recommend to plot squared standardized level-two residuals against 'relevant level-two variables' (covariates with random slopes) (Snijders & Bosker, 2012). Due to convergence failures, none of the models in this study had covariates with random slopes – this assumption is thus not tested here.

6.5 Level-two residual normality

Normal probability plots of standardized level-two residuals were used to check for level-two residual normality (Snijders & Bosker, 2012). Normal Q-Q plots of standardized level-two residuals for random intercepts (due to convergence issues, no models in this study had random slopes) revealed deviations from normality for the random intercepts (see Figure S5.4). However, Maas and Hox (2004) have shown that estimates of fixed effects and their standard errors are robust to non-normal level-two residual errors when there are at least 50 level-two units (this study had at least 150 participants from at least 70 teams at all time points). Given this, it can be assumed that fixed effect estimates (and their standard errors) are unbiased.

6.6 Level-two linearity

To check for level-two linearity, it is recommend to plot unstandardized level-two residuals as a function of relevant level-two variables (covariates with random slopes) (Snijders & Bosker,

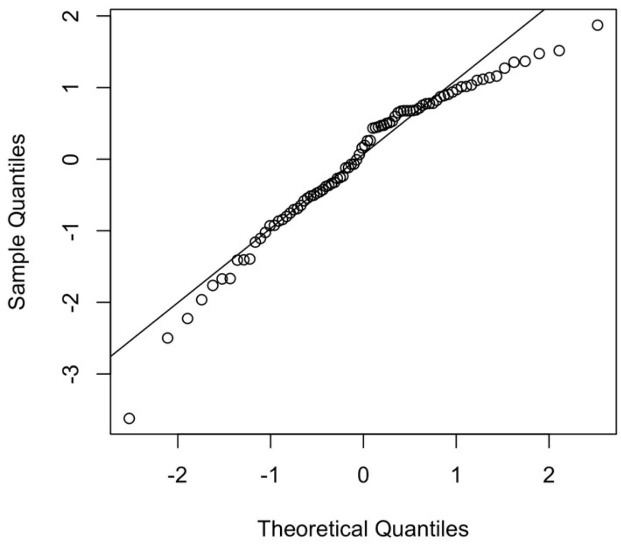

Figure S5.4 Standardized level-two intercept residuals versus the expected order statistics of a normal distribution (Q-Q plot).

2012). Due to convergence failures, none of the models in this study had covariates with random slopes – this assumption is thus not tested here.

References

Freeman, P., Coffee, P., Moll, T., Rees, T., & Sammy, N. (2014). The ARSQ: the athletes' received support questionnaire. *Journal of Sport and Exercise Psychology, 36*(2), 189-202.

Freeman, P., Coffee, P., & Rees, T. (2011). The PASS-Q: The perceived available support in sport questionnaire. *Journal of Sport and Exercise Psychology, 33*(1), 54-74.

Henry, K. B., Arrow, H., & Carini, B. (1999). A tripartite model of group identification theory and measurement. *Small Group Research, 30*(5), 558 - 581. doi:10.1177/104649649903000504

Hu, L. t., & Bentler, P. M. (1999). Cutoff criteria for fit indexes in covariance structure analysis: Conventional criteria versus new alternatives. *Structural equation modeling: a multidisciplinary journal, 6*(1), 1-55.

Maas, C. J., & Hox, J. J. (2004). Robustness issues in multilevel regression analysis. *Statistica Neerlandica, 58*(2), 127-137.

Matsunaga, M. (2010). How to Factor-Analyze Your Data Right: Do's, Don'ts, and How-To's. *International Journal of Psychological Research, 3*(1), 97-110.

Peacock, E. J., & Wong, P. T. P. (1990). The Stress Appraisal Measure (SAM): A multidimensional approach to cognitive appraisal. *Stress Medicine, 6*, 227-236. doi:10.2307/40729741?ref=search-gateway:cebc147346729d5ce5e1fe9ebc09fd2f

Putnick, D. L., & Bornstein, M. H. (2016). Measurement invariance conventions and reporting: The state of the art and future directions for psychological research. *Developmental review, 41*, 71-90.

Raudenbush, S. W., & Bryk, A. S. (2002). *Hierarchical linear models: Applications and data analysis methods* (Vol. 1): Sage.

Revelle, W., & Revelle, M. W. (2015). Package 'psych'. *The comprehensive R archive network, 337*, 338.

Rosseel, Y. (2012). Lavaan: An R package for structural equation modeling and more. Version 0.5–12 (BETA). *Journal of Statistical Software, 48*(2), 1-36.

Seco, G. V., García, M. A., García, M. P. F., & Rojas, P. E. L. (2013). Multilevel bootstrap analysis with assumptions violated. *Psicothema, 25*(4), 520-528.

Snijders, T. A. B., & Bosker, R. J. (2012). *Multilevel analysis: an introduction to basic and advanced multilevel modeling* (2nd ed.): Sage.

Steenkamp, J.-B. E., & Baumgartner, H. (1998). Assessing measurement invariance in cross-national consumer research. *Journal of consumer research, 25*(1), 78-90.

Steinmetz, H. (2013). Analyzing observed composite differences across groups. *Methodology: European Journal of Research Methods for the Behavioral and Social Sciences, 9*, 1-12. doi:10.1027/1614-2241/a000049

Swann, W. B., Gomez, A., Seyle, D. C., Morales, J., & Huici, C. (2009). Identity fusion: the interplay of personal and social identities in extreme group behavior. *Journal of Personality and Social Psychology, 96*(5), 995.

Terry, P. C., Lane, A. M., Lane, H. J., & Keohane, L. (1999). Development and validation of a mood measure for adolescents. *Journal of Sports Sciences, 17*(11), 861-872.

Vandenberg, R. J., & Lance, C. E. (2000). A review and synthesis of the measurement invariance literature: Suggestions, practices, and recommendations for organizational research. *Organizational research methods, 3*(1), 4-70.

Vangelisti, A. L., & Caughlin, J. P. (1997). Revealing family secrets: The influence of topic, function, and relationships. *Journal of Social and Personal Relationships, 14*(5), 679-705. doi:10.1177/1077800406294947

Zumeta, L. N., Oriol, X., Telletxea, S., Amutio, A., & Basabe, N. (2016). Collective Efficacy in Sports and Physical Activities: Perceived Emotional Synchrony and Shared Flow. *Frontiers in Psychology, 6*(1572), 720. doi:10.1086/678698
